# Low Temperature Mitigates Cardia Bifida in Zebrafish Embryos

**DOI:** 10.1371/journal.pone.0069788

**Published:** 2013-07-26

**Authors:** Che-Yi Lin, Cheng-Chen Huang, Wen-Der Wang, Chung-Der Hsiao, Ching-Feng Cheng, Yi-Ting Wu, Yu-Fen Lu, Sheng-Ping L. Hwang

**Affiliations:** 1 Institute of Bioscience and Biotechnology, National Taiwan Ocean University, Keelung, Taiwan; 2 Department of Biology, University of Wisconsin-River Falls, River Falls, Wisconsin, United States of America; 3 Graduate Institute of Agricultural Biotechnology, National Chiayi University, Chiayi, Taiwan; 4 Department of Bioscience Technology, Chung Yuang Christian University, Chungli, Taiwan; 5 Department of Pediatrics, Tzu Chi General Hospital, Hualien, Taiwan; 6 Institute of Cellular and Organismic Biology, Academia Sinica, Nankang, Taipei, Taiwan; Leiden University Medical Center, Netherlands

## Abstract

The coordinated migration of bilateral cardiomyocytes and the formation of the cardiac cone are essential for heart tube formation. We investigated gene regulatory mechanisms involved in myocardial migration, and regulation of the timing of cardiac cone formation in zebrafish embryos. Through screening of zebrafish treated with ethylnitrosourea, we isolated a mutant with a hypomorphic allele of *mil* (*s1pr2*)/*edg5*, called *s1pr2^as10^* (*as10*). Mutant embryos with this allele expressed less *mil*/*edg5* mRNA and exhibited cardia bifida prior to 28 hours post-fertilization. Although the bilateral hearts of the mutants gradually fused together, the resulting formation of two atria and one tightly-packed ventricle failed to support normal blood circulation. Interestingly, cardia bifida of *s1pr2^as10^* embryos could be rescued and normal circulation could be restored by incubating the embryos at low temperature (22.5°C). Rescue was also observed in *gata5* and *bon* cardia bifida morphants raised at 22.5°C. The use of DNA microarrays, digital gene expression analyses, loss-of-function, as well as mRNA and protein rescue experiments, revealed that low temperature mitigates cardia bifida by regulating the expression of genes encoding components of the extracellular matrix (*fibronectin 1*, *tenascin-c*, *tenascin-w*). Furthermore, the addition of N-acetyl cysteine (NAC), a reactive oxygen species (ROS) scavenger, significantly decreased the effect of low temperature on mitigating cardia bifida in *s1pr2^as10^* embryos. Our study reveals that temperature coordinates the development of the heart tube and somitogenesis, and that extracellular matrix genes (*fibronectin 1*, *tenascin-c* and *tenascin-w*) are involved.

## Introduction

During zebrafish cardiac development, heart progenitors are located near the ventral lateral margins at 5 hours post-fertilization (hpf). By 12 hpf, both myocardial and endocardial progenitors appear bilaterally under the future hindbrain region. Bilateral cardiomyocytes then migrate toward the midline and fuse to form a cone-shaped heart at the 21-somite stage (ss). Next, the heart cone begins to elongate, and forms a single heart tube at 24 hpf [1]–[3].

Previous studies have demonstrated that mutations in several genes cause defective migration of myocardial precursors, including genes involved in myocardium formation [4], [5], endoderm formation [5]–[7], extracellular matrix composition [8], and sphingosine-1-phosphate signaling [9]–[11]. Studies on *fibronectin* mutants of mice and chick embryos incubated with fibronectin antibodies demonstrated that fibronectin is the major extracellular matrix component that directs myocardial migration to the midline [12], [13]. In zebrafish, fibronectin has been shown to be required for the proper formation of adherens junctions between myocardial precursor cells; this in turn maintains epithelial integrity, which is essential for myocardial migration [8]. Increased levels of *fibronectin 1* (*fn1*) mRNA were previously observed in *hand2* mutants; a genetic reduction of *fn1* gene dosage in *hand2* mutants partially rescued the cardia bifida phenotype, thereby implicating fibronectin in the development of this disorder [14].

The *mil/edg5* gene encodes a G protein-coupled receptor which binds to sphingosine-1-phosphate in the lateral plate mesoderm [10], while *toh/spns2* encodes the sphingosine-1-phosphate transporter in the yolk syncytial layer, required for transport of sphingosine-1-phosphate to the mesoderm [9], [11]. Mutations in either gene have been reported to cause cardia bifida. Injection of fibronectin into the midline of *mil* 14-ss embryos could partially rescue the cardia bifida, and caused partial fusion of bilateral cardiomyocytes [15]. Decreased levels of fibronectin were also detected at the midline in embryos injected with *edg5-*morpholino antisense oligomers (MO) [11]. The findings of these studies indicate that the cardia bifida exhibited by *mil* mutant embryos can be partially attributed to decreased cell-fibronectin interactions. However, both the regulatory mechanisms that control genes involved in myocardial migration, and the developmental processes required for heart tube formation, remain unclear.

In this study, we screened zebrafish treated with ethylnitrosourea (ENU), and isolated a hypomorphic allele of *mil* (*s1pr2*)/*edg5*, designated as *s1pr2^as10^* (*as10*). Embryos with the *s1pr2^as10^* allele exhibited decreased *mil*/*edg5* expression, and this was associated with cardia bifida at 24 hpf. Interestingly, the cardia bifida phenotype of *s1pr2^as10^* embryos could be rescued (restoring normal circulation) by incubating the embryos at a low temperature (22.5°C). A similar rescue was observed in *gata5* and *bon* cardia bifida morphants raised at 22.5°C. DNA microarray, MO knockdown, and rescue experiments with mRNA or protein revealed that low temperature mitigates cardia bifida by regulating the expression of extracellular matrix genes, particularly in the anterior lateral plate mesoderm, midline, and pharyngeal arch regions.

## Materials and Methods

### Ethics Statement

All animal procedures were approved by the Animal Use and Care Committee of Academia Sinica (Protocol # RFiZOOHS2010065).

### Zebrafish Maintenance and Staging, and Low Temperature and N-acetyl Cysteine (NAC) Treatment

Adult zebrafish strains, including AB, SJD, *Tg*(*−7.5bmp4:GFP*)*^as1^*, *Tg*(*cmlc2:EGFP, cmlc2:H2AFZmCherry*)*^cy13^*, *s1pr2^as10^* (*as10*), and *mil* (*s1pr2^m93/+^*)/*edg5*, were raised under standard conditions, as previously described [16]. To perform the rescue experiments, embryos were incubated at 22.5°C from the one-cell zygote stage onwards. Two staging systems for zebrafish embryos were used in this study, the somite stage (ss), which is determined by counting the somite number, and the conventional temporal stage, which is determined by hours post-fertilization (hpf). We also followed the previously described morphological criteria for staging [17]. One-cell zygotes of *as10* were incubated at 22.5°C and treated with 50 or 150 µM N-acetyl cysteine (NAC) (Merck) from the tailbud stage onwards. NAC-treated embryos were continuously incubated at 22.5°C and allowed to develop to the 26 ss stage; the position of migrating cardiomyocytes was examined at this stage.

### Mutagenesis and Isolation of *s1pr2^as10^* Mutants

Random mutagenesis was conducted using *Tg*(*−7.5bmp4:GFP*)*^as1^* transgenic fish, as previously described [18]. ENU-treated *Tg*(*−7.5bmp4:GFP*)*^as1^* males were outcrossed to untreated homozygous *Tg*(*−7.5bmp4:GFP*)*^as1^* females to generate a larger stock of F1 founders, and mutant screening was performed using the early pressure protocol to generate parthenogenic homozygous F2 embryos [18].

### Positional Cloning for *s1pr2^as10^*

*s1pr2^as10^* heterozygous mutant fish in the AB background were outcrossed to the wild-type SJD background to generate mapping stocks. Simple sequence length polymorphism (SSLP) markers were used to perform the linkage analyses. For the low-resolution mapping analysis, *s1pr2^as10^* was mapped to chromosome 3 between markers z3725 (9 recombinants out of 71 meioses, 9/71) and Z39291 (2/78). To construct a high-resolution map around the *s1pr2^as10^* locus, 900 *s1pr2^as10^* homozygous mutant embryos and custom-designed SSLP markers were used. The minimum recombinants included markers CU57-40022 (1/900) and CR38-83127 (2/900). The sequences of the primer pair for CU57-40022 were F-TCATATCGATTGACCACCCTAA and R-ATTATGGAGCAGCCGAGCTA. The sequences of the primer pair for CR38-83127 were F-GTCAGTGTCACTCCCCTGGT and R-TGTGACCCATTCTTTCATTTCTT.

### Ligation-mediated Polymerase Chain Reaction (PCR)

Ligation-mediated PCR was performed to identify an insertion in intron 1 of the *mil*/*edg5* gene. Genomic DNA isolated from *s1pr2^as10^* mutants was digested with *Nla*III, and ligated to an Nla linker prepared by annealing Nla oligo 1 (GTAATACGACTCACTATAGGGCTCCGCTTAAGGGACCATG) and Nla oligo 2 (GTCCCTTAAGCGGAG). The first PCR was conducted using the Nla linker-ligated genomic DNA of *s1pr2^as10^* mutants as a template with primer 1 (GTAATACGACTCACTATAGGGC) and primer 2 (AGAGTGGTCATGTGGTGGTC). A nested PCR reaction was subsequently performed using a 1∶50 dilution of the first PCR product as a template with primer 1 (AGGGCTCCGCTTAAGGGAC) and primer 2 (CTGATAAGTAAATGAATGAACACA). The candidate PCR products were subsequently gel-eluted and cloned into the pGEM-T vector (Promega). Sequence analyses indicated that the insertion was derived from chromosome 11. To further confirm the presence of the insertion in intron 1 of the *mil*/*edg5* gene, genomic DNA was isolated from wild-type, the wild-type siblings of *s1pr2^as10^*, and *s1pr2^as10^* mutant embryos, and used as templates for PCR with primers either flanking or within the insertion (Figure S1B). The sequences of the three primers were as follows: F1-AAAACATCTGCACGCGCTTCTTC; R1-ATAAGAGTGGTCATGTGGTGG TC; and I1-GCGTGGCTCATTATACTTCAGGA.

### Quantitative Real-time Reverse-transcription Polymerase Chain Reaction (qRT-PCR)

qRT-PCR was conducted using 2x SYBR green PCR master mix (Roche) with a Roche Light Cycler 480 II thermal cycler. The following genes were amplified using the indicated primers: *edg5* (F- GCAGCAGTCACTCCGCAAAAG and R- ACGC CCAGCCCGAAGTCAC); *fn1* (F- GACACGGCCCACAGAGACTAT and R- TGG GCGTGATTTTACAGGTG); *tnc* (F- TTGGAGAAGGCCGGTTGCTAAAAT and R- CAGGGTTCAGGCCAGTCAGGATG); *tnw* (F- CAGTGGGAGCAGCAGGCAG AC and R- GTATGGACGTTGTGGATTTCAGTA); *β-actin* (F-CGAGCAGGAGA TGGGAACC and R-GGGCAACGGAAACGCTCAT).

### Morpholino Antisense Oligomer (MO)-mediated Knockdown

The sequences of MOs used in this study include: *bon*-MO [19] (GACTGCCATTGTGCTGCTGTCCTTC); *edg5*-MO [20] (AGACGGCAAGTAGTCATTCAGAGGG); *edg5*-5 mm (AGtCcGCAAcTAcTCATTCAcAGGG); *fn1*-MO [8] (TTTTTTCACAGGTGCGATTGAACAC); *gata5*-MO [21] (TGTTAAGATTTTTACCTATACTGGA); *tnc*-MO1 [22] (GAGAGGATCTCACAGGACACTCC); *tnc*−5mm (GAcAcGATgTCACAGcAgACTCC); *tnc-*MO2 [22] (TATATGGGCTCACCTGTAACCTGAG); *tnw*-MO1 (AATATTCCCTGCCACAGTAACTTTC); and *tnw*−5mm (AATAaTgCCTcCCACAcTAAgTTTC); *tnw-*MO2 (ACGTCTATTACTGCAAGCACCTGTT) (Gene Tools). MOs or mRNA synthesized *in vitro* were individually microinjected into one- or two-cell zygotes, using an IM-300 microinjector (NARISHIGE). Human fibronectin (1 mg/mL; Sigma) and Tenascin-C (0.1 mg/mL; Millipore) protein were individually microinjected into the midline region at 14–16 hpf (10–14 ss).

### Whole-mount *in Situ* Hybridization, Histological Analysis, and Photography

Embryos treated with 0.003% phenylthiocarbamide were subjected to whole mount *in-situ* hybridization, using digoxigenin- or fluorescein-labeled antisense RNA probes and either alkaline phosphatase-conjugated anti-digoxigenin or anti-fluorescein antibodies, as previously described. Various templates derived from the pGEM-T vector were linearized, and the following antisense RNA probes were generated, using the restriction sites and promoters in parentheses: *amhc* (*Nco* I/sp6), *cmlc2* (*Nco* I/sp6), *foxa1* (*Sal* I/T7), *fn1* (*Nco* I/sp6), *tnc* (*Apa* I/sp6), and *tnw* (*Sac* II/sp6). Paraffin sectioning (5 µm) and haematoxylin and eosin staining were performed using standard procedures. All images were taken using a Zeiss AxioCam HRC camera mounted on a Zeiss Imager M1 microscope.

### Immunohistochemistry

Immunohistochemistry was performed as previously described [23]. In brief, embryos were fixed in 2% paraformaldehyde at 4°C overnight. Fixed embryos were embedded in 4% low-melting point agarose and sectioned with a vibratome (Leica VT1000M) to sections of 200 µm. The sections were washed with 1% Triton X-100 in PBS (PBSTx) at room temperature, before being incubated in blocking solution (10% lamb serum in PBSTx). Sections were then incubated with an anti-fibronectin antibody (Sigma) at a dilution of 1∶100 for 1.5 days at 4°C. Alexa Fluor 488 goat anti-rabbit IgG (Invitrogen) was used as a secondary antibody, at a dilution of 1∶200. The sections were washed with blocking solution and PBSTx at room temperature. After staining, sections were examined with a Leica Confocal Microscope (TCS SP5 MP).

### DNA Microarray, Gene Ontology (GO), and Pathway Analyses

Total RNA was extracted from 22-ss wild-type and *s1pr2^as10^* mutant embryos raised at either 28.5 or 22.5°C using an RNeasy Plant Mini Kit (Qiagen), and 10 µg of RNA was treated with RNase-free DNase I. The RNA quality was verified with a Bioanalyzer 2100 (Agilent). DNA microarray analysis was performed by NimbleGen using Zebrafish Gene Expression 12×135K arrays (NimbleGen) containing 38,489 genes. The 1,844 genes that exhibited a 1.3-fold or greater change in expression between embryos raised at 28.5 and 22.5°C for either *s1pr2^as10^* homozygous mutants or wild-type were clustered into 5 groups using GeneSpring GX software (Agilent). The same strategy was applied to the 264 genes that exhibited a 1.5-fold or greater change in expression. The identified genes were subjected to gene ontology (GO) analysis using a Batch-Genes tool based on a zebrafish or human database (GOEAST, http://omicslab.genetics.ac.cn/GOEAST/tools.php), for which the *p* values were smaller than 0.1 [24]. Pathway analysis was performed on these genes using the website of the Database for Annotation, Visualization and Integrated Discovery (DAVID, http://david.abcc.ncifcrf.gov/), for which *p* values were smaller than 0.1 [25], [26].

### Digital Gene Expression (DGE) Analysis

Total RNA was extracted from 22-ss *s1pr2^as10^* mutant embryos raised at 28.5 or 22.5°C using an RNeasy Plant Mini Kit (Qiagen), and 4 µg was treated with RNase-free DNase I. The RNA quality was verified with a Bioanalyzer 2100 (Agilent). DGE analysis was performed by BGI through next-generation sequencing (NGS). In brief, Oligo(dT) beads were used to enrich mRNA from total RNA, and the mRNA was then reverse transcribed into double-stranded cDNA, using reverse transcriptase and DNA polymerase. The cDNA was digested with *Nla*III and ligated to Illumina adaptor 1. The cDNA was then cut at 17 bp downstream of the CATG site using *Mme*I, and its 3′ end was ligated to Illumina adaptor 2. PCR was performed using primers GX1 and GX2, which target adaptors 1 and 2, respectively. The resulting 95-bp fragments were isolated by 6% TBE polyacrylamide gel electrophoresis (PAGE), and the DNA was purified and subjected to Illumina sequencing using a Genome Analyzer II (Illumina).

## Results

### Phenotypic Characterization of the *s1pr2^as10^* Mutation

**Figure 1:**
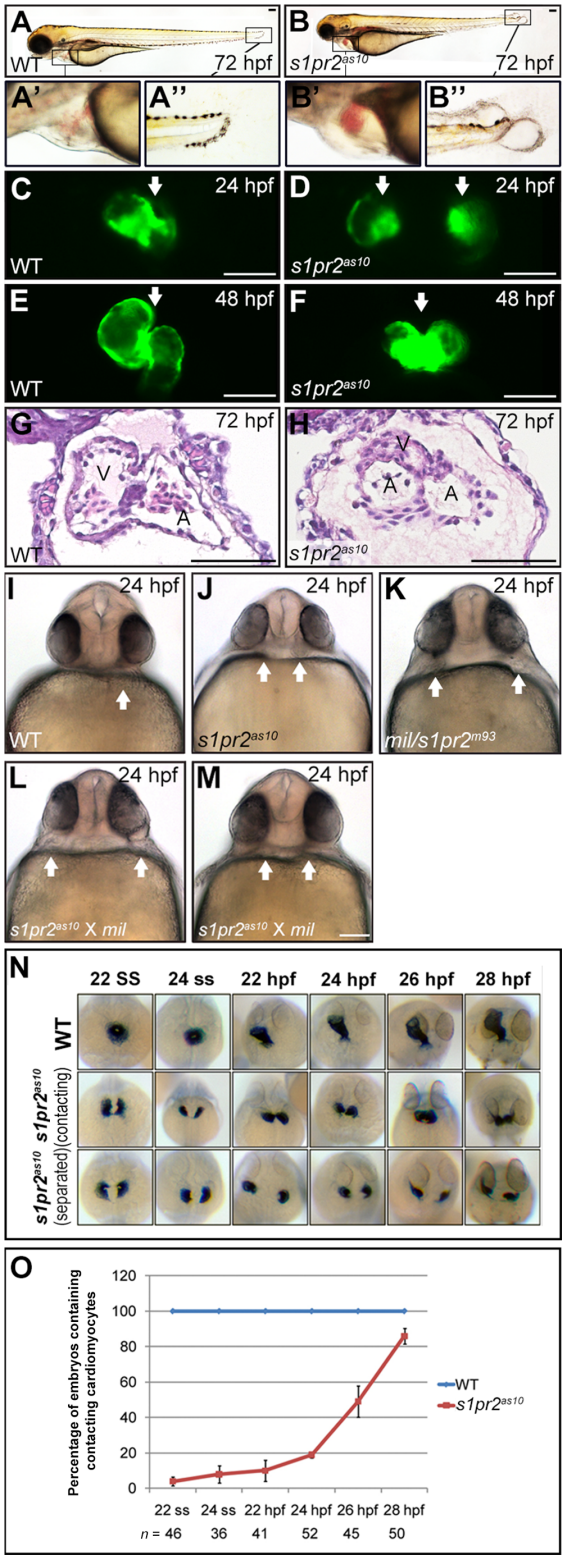
The phenotype of the *s1pr2^as10^* mutant. (A-H) Lateral views of wild-type (WT) (A, C, E and G) and *s1pr2^as10^* mutants (B, D, F and H). Pericardial edema (B’), small eyes, and tail blisters (B’) were observed in 72-hpf *s1pr2^as10^* mutant embryos (B). Two separate hearts (white arrows, D) were detected at 24 hpf, whereas a fused heart (white arrow, F) was observed at 48 hpf in *s1pr2^as10^* mutant embryos. Paraffin sectioning with haematoxylin and eosin staining revealed the presence of two atria and one ventricle in *s1pr2^as10^* mutant embryos at 72 hpf (H). (I-M) Ventral views of wild-type (WT) (I), *s1pr2^as10^* (J), *mil* (K), and the intercross mutant progeny of *s1pr2^as10^* and *mil* mutants (L and M) at 24 hpf are shown. The arrows indicate the positions of the hearts. (N) Cardiomyocytes were labeled via *cmlc2* staining. Two representative *s1pr2^as10^* mutant embryos are shown. Both contacting and separated cardiomyocytes were detected in the *s1pr2^as10^* mutant embryos from the 22 ss to 28 hpf. (O) Percentages of WT and *s1pr2^as10^* mutants containing contacting myocardia at different developmental stages. Scale bar  = 100 µm. The error bars indicate the standard error.

In order to screen for mutants with heart morphogenetic defects, we conducted a ethylnitrosourea mutagenesis screen of the *Tg*(*−7.5bmp4:GFP*)*^as1^* transgenic zebrafish line, which expresses GFP in the heart after 24 hpf. Through this screen, we isolated the *s1pr2^as10^* mutant line (Figure 1B), which is a recessive embryonic lethal mutation with several phenotypes, including pericardial edema (Figure 1 B’), small eyes, and tail blisters (Figure 1 B’). Blood circulation was not established during the embryonic and larval periods. We subsequently crossed *s1pr2^as10^* into a *Tg*(*cmlc2:EGFP, cmlc2:H2AFZmCherry*)*^cy13^* background to generate higher fluorescence intensity in the bilateral cardiomyocytes. In contrast to wild-type (WT) myocardial precursors, which migrated toward the midline and fused to form a single heart tube by 24 hpf (Figure 1C), myocardial precursors in the *s1pr2^as10^* mutants remained in the bilateral lateral plate mesoderm (LPM) at 24 hpf (Figure 1D). Although the *s1pr2^as10^* bilateral myocardial precursors eventually reached the midline, they formed a three-chambered heart with two atria and one tightly-packed ventricle from 48 hpf (Figure 1F and 1H); this is in contrast to WT hearts, which contain a single atrium and ventricle (Figure 1E and 1G). These results indicate that the newly identified *s1pr2^as10^* mutation delays the fusion of bilateral myocardiocytes, leading to the formation of non-functional three-chambered hearts.

### Genetic Mapping of the *s1pr2^as10^* Mutant Gene

By performing positional cloning with simple sequence length polymorphism markers, we established that *s1pr2^as10^* is located on chromosome 3 near the *mil/edg5* locus (Figure S1A). Sequencing of the *mil/edg5* coding regions within cDNA or genomic DNA of *s1pr2^as10^* mutant embryos did not predict any changes in the amino acid sequence (data not shown). Next, we examined the non-coding regions of the *mil/edg5* locus via ligation-mediated polymerase chain reaction (PCR) and identified a large insertion (more than 1,000 base pairs from linkage group 11) in the first intron of *mil/edg5*, near exon 1 (data not shown). To confirm this insertion, we performed PCR with three primers, including F1 and R1 outside the insertion and I1 within the insertion (Figure S1B and S1C). A 426*-*bp DNA fragment amplified by primers I1 and R1 was detected only in the *s1pr2^as10^* heterozygote and homozygote mutant embryos. To examine the impact of the insertion on *mil/edg5* gene expression, we performed quantitative real-time reverse-transcription polymerase chain reaction (qRT-PCR) to compare the expression levels of *mil/edg5* between the *s1pr2^as10^* mutants and their WT siblings during and after cardiogenesis. Significantly reduced *mil*/*edg5* mRNA levels were detected in the *s1pr2^as10^* mutants prior to 96 hpf (Figure S1D). These results strongly suggest that the *s1pr2^as10^* cardiac defect is caused by hypomorphic *mil*/*edg5* gene expression and that *s1pr2^as10^* may represent a new allele of *mil*/*s1pr2^m93^*. Complementation experiments further demonstrated that *s1pr2^as10^* failed to complement a *mil* mutant at 24 hpf (Figure 1L and 1M vs. 1K). In summary, *s1pr2^as10^* is a hypomorphic allele of *mil* that is caused by an insertion into intron 1.

### *s1pr2^as10^* Mutants Exhibit a Weaker Phenotype than *mil* Mutants

Next, we carefully compared the cardiac phenotypes of *s1pr2^as10^* and *mil* mutations. We observed that the average distance between bilateral hearts at 24 hpf in *s1pr2^as10^* mutant embryos was shorter than that of *mil* mutants (Figure 1J and 1K). In addition, while the bilateral hearts persisted until at least 48 hpf in *mil* mutants, the myocardia fused by 48 hpf in *s1pr2^as10^* mutants (Figure 1F). Both of these observations indicate that the cardia bifida of *s1pr2^as10^* mutants is weaker than that of *mil* mutants. We subsequently conducted time-lapse analyses of *cmlc2-*stained embryos to investigate the timing of bilateral heart fusion in the *s1pr2^as10^* mutant (Figure 1N). At 24 hpf, the majority of *s1pr2^as10^* mutant embryos contained two clusters of laterally positioned myocardial precursors, whereas by 28 hpf, over 80% of mutant embryos contained myocardial precursors in contact with one another (Figure 1O). To demonstrate that the *s1pr2^as10^* mutant phenotype could be attributed to the low levels of *mil*/*edg5* mRNA, we injected different doses of *edg5*-MO or *edg5*-5mm MO into *Tg*(*cmlc2:EGFP, cmlc2:H2AFZmCherry*)*^cy13^* transgenic fish at the one-cell zygote stage. To quantitatively assess the severity of cardiac abnormalities, we grouped the cardiac phenotypes into three classes; Class I represented a normal heart tube, Class II represented cardiomyocytes either in close proximity or in contact, and Class III represented the most severe cardia bifida (Figure S2A). At 24 hpf, dose-dependent cardia bifida was detected in embryos injected with 1 to 16 ng of *edg5*-MO, but not in embryos injected with *edg5*-5mm (Figure S2B). Injection of *s1pr2^as10^* mutant embryos with 30 pg *mil*/*edg5* mRNA resulted in a partial rescue of cardia bifida at 24 hpf, as compared to mutant embryos injected with *LacZ* (Figure S3). Overall, these data indicate that the hypomorphic migration defect of myocardial precursors in *s1pr2^as10^* mutant embryos was a result of decreased levels of *mil/edg5* mRNA.

### Raising *s1pr2^as10^* Embryos at low Temperature Rescued the Cardiac Phenotype

**Figure 2:**
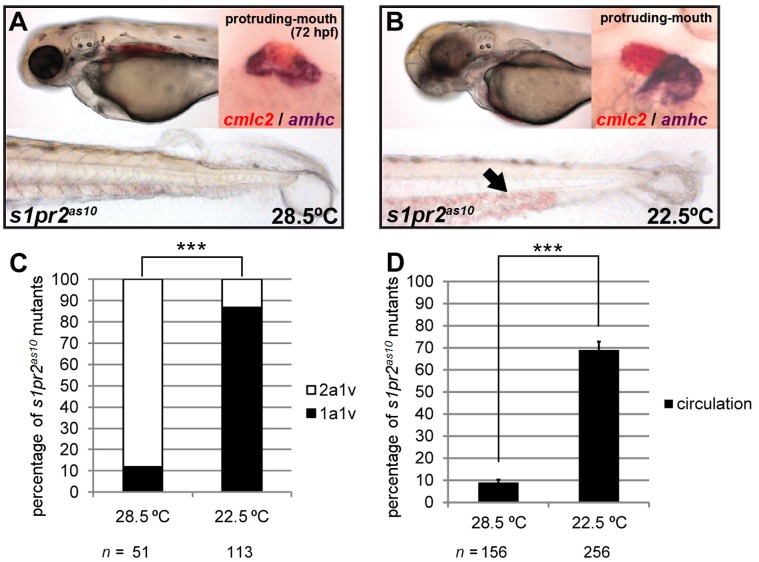
Development at low temperature rescues the *s1pr2^as10^* cardiac phenotype. (A) *s1pr2^as10^* mutants raised at 28.5°C contained hearts with two atria (dark purple, *amhc* staining) and one ventricle (red, *cmlc2* staining), and presented with tail blisters at 72 hpf. (B) *s1pr2^as10^* mutant embryos raised at 22.5°C contained hearts with a single atrium and ventricle, and exhibited blood circulation at 72 hpf (arrow). (C) Percentages of *s1pr2^as10^* mutant embryos raised at either 28.5 or 22.5°C containing two atria and one ventricle (2a1v) or one atrium and one ventricle (1a1v) at 72 hpf. (D) Percentages of *s1pr2^as10^* mutant embryos raised at either 28.5 or 22.5°C displaying blood circulation at 72 hpf. The error bars indicate the standard error. Statistical significance was determined using Student’s *t*-test. *** indicates *p*<0.001.

The hearts of *s1pr2^as10^* mutant embryos raised at a standard temperature (28.5°C) contained two atria and one tightly-packed ventricle, as visualized by *cmlc2* and *amhc* double-staining; most of these embryos lacked blood circulation at 72 hpf (Figure 2A). However, we found that the *s1pr2^as10^* mutants raised at a lower temperature (22.5°C) presented with a less severe phenotype (Figure 2B). More than 85% of the *s1pr2^as10^* mutant embryos raised at 22.5°C had hearts with one atrium and one ventricle at 72 hpf, as compared to only 12% of the embryos raised at 28.5°C (Figure 2C). In addition, normal blood circulation was detected at 72 hpf in 69% of the *s1pr2^as10^* mutant embryos raised at 22.5°C, but in only 9% of those raised at 28.5°C (Figure 2D). Rescued *s1pr2^as10^* homozygous mutants grew to adulthood. A high proportion of the offspring of rescued mutants were also rescued when raised at 22.5°C (Table S1). However, the tail blister phenotype of the *s1pr2^as10^* mutant embryos could not be rescued by development at 22.5°C (Figure 2B). This result indicates that incubation at low temperature specifically affects myocardial migration, and that *s1pr2^as10^* is not simply a temperature-sensitive allele of *mil*/*edg5*. We subsequently performed temperature shift experiments, by incubating *s1pr2^as10^* mutants from different stages at 28.5°C or 22.5°C. We found that the critical time window in which low temperature could rescue cardia bifida in *s1pr2^as10^* mutants was from ∼16 to the 22 somite stage (ss) (Figure S4).

### Low Temperature Shifts the Timing of Cardiac Cone Formation

**Figure 3:**
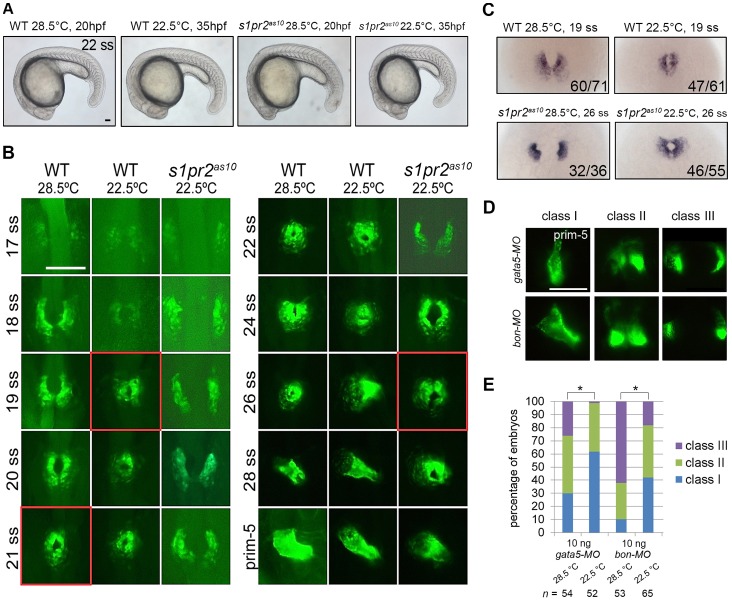
Low temperature shifts the timing of formation of the cardiac cone. (A) Lateral views of 22-ss wild-type (WT) and *s1pr2^as10^* mutant embryos raised at 28.5 or 22.5°C. (B) Time-lapse analyses of the medial migration of bilateral cardiomyocytes during the ∼17–30-ss in *Tg*(*cmlc2:EGFP, cmlc2:H2AFZmCherry*)*^cy13^* transgenic fish (WT) or *s1pr2^as10^* mutant embryos raised at 28.5 or 22.5°C. Five WT or *s1pr2^as10^* mutant embryos were analyzed for each stage. The images outlined in red indicate the stage at which cardiac cone formation took place under each condition. (C) *In situ* hybridization of 19-ss wild-type (WT) or 26-ss *s1pr2^as10^* mutant embryos raised at 28.5°C or 22.5°C with a *cmlc2* RNA probe. The number of embryos displaying *cmlc2* staining/the total number of embryos analyzed is shown for each panel. (D) Different degrees of myocardial migration defects were observed in *gata5* and *bon* morphants at 24 hpf. Class I (single heart tube), Class II (cardiomyocytes in close proximity but not in contact), and Class III (two separate hearts) defects are shown. (E) Percentages of each class of myocardial migration defects in 10 ng *gata5*-MO or *bon*-MO-injected embryos raised at 28.5 or 22.5°C. Scale bar  = 100 µm. Statistical significance was determined using Student’s *t*-test. * indicates *p*<0.05.

To examine why the cardiac defects of the *s1pr2^as10^* mutants were rescued by low temperature, we conducted time-lapse experiments to observe bilateral myocardial precursor migration at different somite stages. In *Tg*(*cmlc2:EGFP, cmlc2:H2AFZmCherry*)*^cy13^* transgenic embryos raised at 28.5°C, the bilateral myocardial precursors fused at the midline and became cone-shaped at the 21-ss (Figure 3B, red boxes in WT at 28.5°C). Surprisingly, the timing of cardiac cone formation was shifted to the 19 ss when the embryos were raised at the lower temperature (Figure 3B, red boxes in WT at 22.5°C). Although the overall growth rate at 22.5°C was about 2-fold slower than that at 28.5°C [27], low temperature does not seem to cause any morphological defects in WT and *s1pr2^as10^* embryos (Figure 3A). Similarly, the bilateral cardiomyocytes in the *s1pr2^as10^* mutant embryos fused at the 26 ss at low temperature (Figure 3B, red boxes in *s1pr2^as10^* 22.5°C); this result was in contrast to the delayed fusion (28 hpf) observed at 28.5°C (Figure 1O). *In situ* hybridization using a *cmlc2* probe revealed that 77% WT and 84% *s1pr2^as10^* mutant embryos raised at low temperature formed a fused cardiac cone at the 19 ss and 26 ss, respectively (Figure 3C). In contrast, the majority of WT and *s1pr2^as10^* mutant embryos raised at standard temperature presented with horseshoe-shaped hearts or bilateral hearts at 19 ss and 26 ss, respectively (Figure 3C). However, cardia bifida of *mil* mutant embryos cannot be rescued by low temperature treatment (Figure S5A). Since defects in endoderm formation also result in cardia bifida [7], [28], we induced cardia bifida by injecting different doses of *gata5* MO or *bon* MO. We found that the cardia bifida phenotypes of embryos injected with 10 ng of *gata5* MO or *bon* MO can be significantly rescued by being raised at low temperature (Figure 3D and 3E) while cardia bifida defects in embryos injected with 20 ng of *gata5* MO were not rescued by low temperature treatment (Figure S5B). Although the rescue effect was not statistically significant, a reduced percentage of cardia bifida phenotype was notable in embryos injected with 20 ng of *bon* MO. (Figure S5B). In addition to cardia bifida, cells of the anterior gut tissues also failed to migrate to the midline in *gata5*/*fau* mutants [28]. We found that the distance between the two lateral anterior gut tubes was also significantly reduced in embryos injected with 10 ng *gata5* MO and incubated at low temperature, as compared to those incubated at standard temperature (145 µm vs. 160 µm) at the prim-25 stage (Figure S6). Thus, low temperature alters the timing of cardiac cone formation in WT, *s1pr2^as10^* mutants (which exhibit hypomorphic migration defects) and other cardia bifida morphants, resulting in a rescue of the cardia bifida phenotype that is independent of the genetic background.

### Gene Expression Profiles of Zebrafish Embryos Raised at 28.5 and 22.5°C

**Figure 4:**
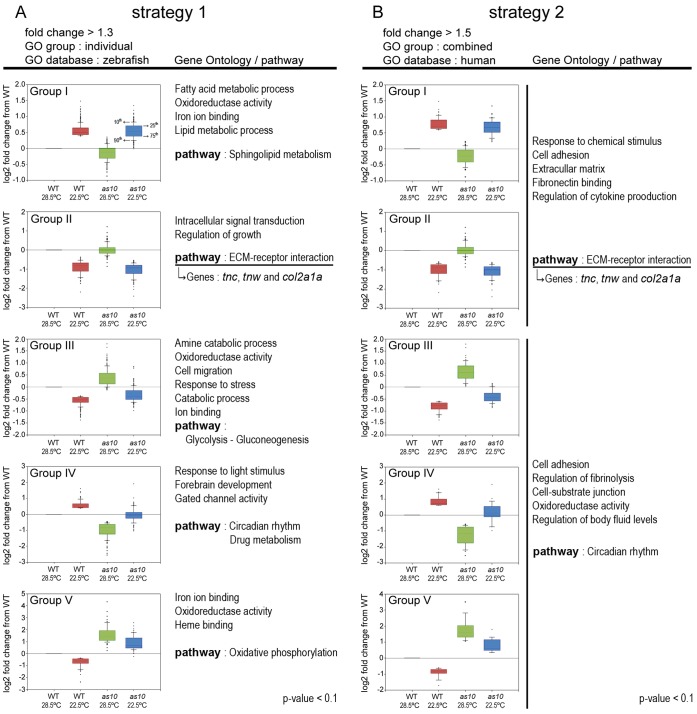
Gene ontology (GO) and pathway analyses for differentially expressed genes identified by DNA microarray analysis. GO and pathway analyses were conducted on different groups of genes using two strategies. (A) Genes with a greater than 1.3-fold change in expression between embryos raised at 28.5 and 22.5°C were used in strategy 1. GO analysis was performed using the zebrafish database. (B) Genes with a greater than 1.5-fold change in expression between embryos raised at 28.5 and 22.5°C were used in strategy 2. GO analysis was performed using the human database. The GO and pathway analyses were performed on genes from both Groups I and II or on genes from Groups III, IV and V. Box and Whisker plots for each group indicate the median (line within the box), 25^th^ and 75^th^ percentiles (top and bottom of the box), and 10^th^ and 90^th^ percentiles (Whiskers/error bars). The P-value from GO and pathway analyses are smaller than 0.1.

Previous reports have demonstrated that changes in temperature affect developmental processes and gene expression in zebrafish embryos [29], [30]. We performed DNA microarray (Figure 4) and digital gene expression (DGE) (Table S2) analyses to further investigate how low temperature mitigates cardia bifida. By combining these two analyses, we identified several candidate gene groups and demonstrated that genes encoding extracellular matrix (ECM) proteins are important for preventing cardia bifida. DNA microarray and qRT-PCR revealed that *mil*/*edg5* levels were not affected by raising WT or *s1pr2^as10^* embryos at different temperatures (28.5°C or 22.5°C) (Figure S7).

### Low Temperature Mitigates Cardia Bifida by Altering ECM Gene Expression

**Figure 5:**
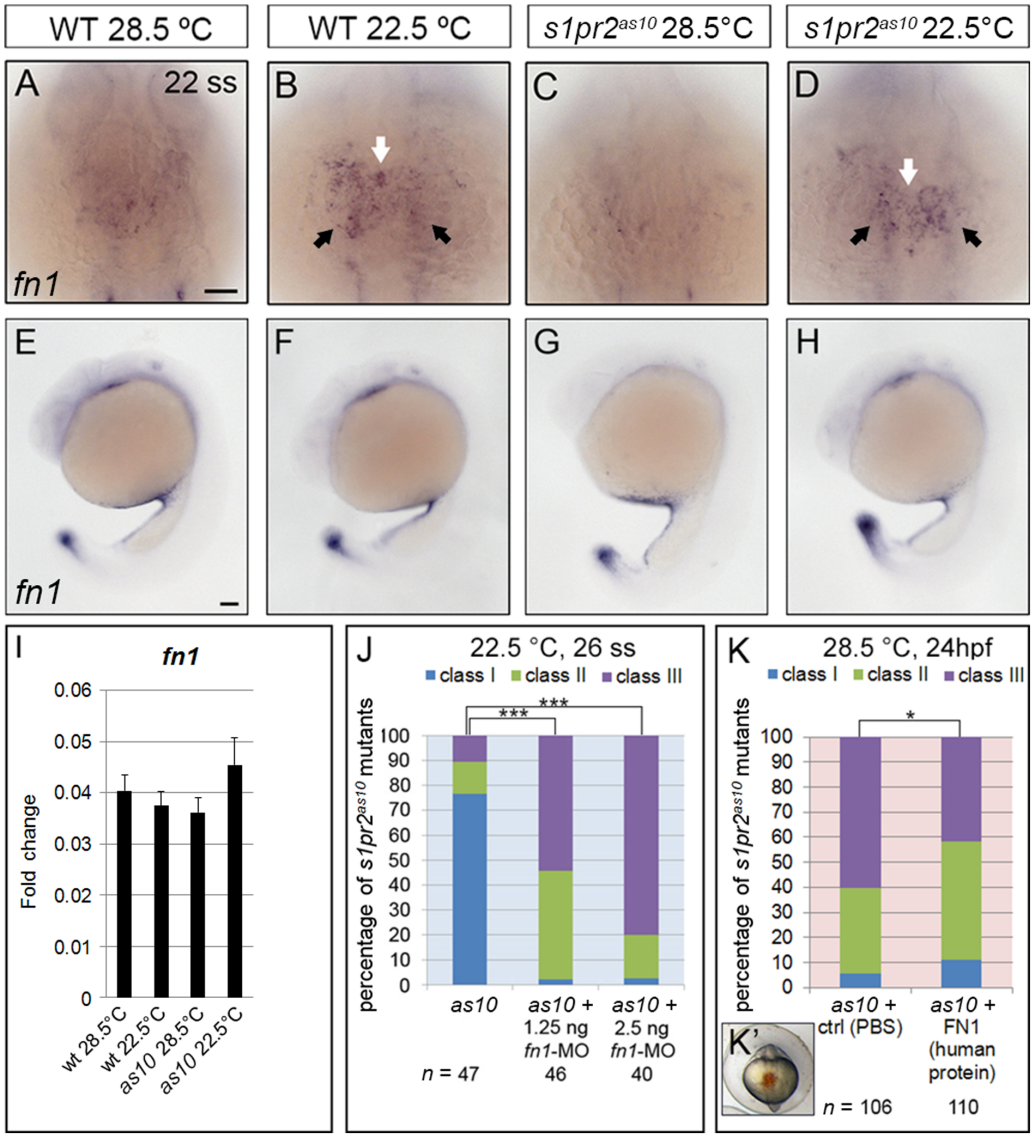
Expression of *fibronectin 1* is affected by temperature. (A-H) *In situ* hybridization against *fibronectin 1* (*fn1*) in 22-ss wild-type (WT) (A, B, E, F) and *s1pr2^as10^* (*as10*) mutant (C, D, G, H) embryos raised at 28.5 or 22.5°C. Expression of *fn1* mRNA increased at the midline region (white arrows in B and D) and bilateral LPM (black arrows in B and D) of both WT and *s1pr2^as10^* mutant embryos raised at 22.5°C, as compared to those raised at 28.5°C (A and C). Expression of *fn1* at the tail bud or yolk regions was not affected (E-H). (I) qRT-PCR revealed a trend towards increased expression of *fn1* mRNA in *s1pr2^as10^* mutant embryos raised at 22.5°C. The error bars indicate the standard error. (J) Knockdown of *fn1* expression in *s1pr2^as10^* mutants increased the percentage of 26-ss embryos raised at 22.5°C with the Class III cardia bifida phenotype. (K) Injection of human fibronectin protein into *s1pr2^as10^* mutant embryos raised at 28.5°C partially rescued the cardia bifida phenotypes at 24 hpf. (K’) The red dye indicates the protein injection region at 14–16 hpf. Scale bars  = 100 µm. Statistical significance was determined using Student’s *t*-test. * indicates *p*<0.05, *** indicates *p*<0.001.

Based on the DNA microarray and DGE analyses, we established that several genes involved in the ECM were affected by low temperature; these genes included *tenascin-c* (*tnc*), *tenascin-w* (*tnw*), and *collagen type II, alpha-1a* identified through the DNA microarray analysis (Figure 4), and *fibronectin 1* (*fn1*), *collagen, type XI alpha 2* and *sid4* through the DGE analysis (Table S2). We first focused on *fn1*, which was previously found to be important for myocardial migration [8]. An increased level of *fn1* was initially identified via DGE in *s1pr2^as10^* mutant embryos raised at 22.5°C (Table S2). Whole mount *in situ* hybridization and immunocytochemistry revealed increased expression of *fn1* mRNA and protein in the midline regions and bilateral anterior LPM of both 22-ss WT and *s1pr2^as10^* mutant embryos raised at 22.5°C (Figure 5B and 5D and Figure S8). However, levels of *fn1* at the tail bud or yolk regions were not affected by incubation at low temperature (Figure 5E–5H). qRT-PCR revealed an approximately 1.3-fold increase of *fn1* mRNA in *s1pr2^as10^* mutant embryos raised at 22.5°C, as compared to those raised at 28.5°C. However, this temperature-mediated up-regulation was not statistically significant (Figure 5I). The failure to detect a significant change in WT and *s1pr2^as10^* mutant embryos raised at low temperature may be due to the inability of temperature to affect *fn1* expression at the tail bud and yolk regions (Figure 5E–5H).

As incubation at low temperature raised *fn1* expression and rescued the cardia bifida phenotype in *s1pr2^as10^*, we hypothesized that *fnl* up-regulation may be required for the rescue of heart development. To test this hypothesis, we injected *fn1*-MO into *s1pr2^as10^* mutants raised at 22.5°C. As shown in Figure 5J, 26-ss mutant embryos injected with 1.25 or 2.5 ng *fn1*-MO displayed severe cardia bifida, even after being raised at the low temperature. In addition, we injected human fibronectin protein into the midline region (Figure 5K’) of 14–16 hpf *s1pr2^as10^* mutant embryos raised at 28.5°C, to determine whether elevated levels of fibronectin can rescue the cardia bifida phenotype. We found that the percentage of mutant embryos with class III severe cardia bifida was reduced from 60% to 42% (Figure 5K). Together, these results indicate that incubation at low temperature mitigates cardia bifida in *s1pr2^as10^* mutants by promoting *fn1* expression in the midline and bilateral anterior LPM regions.

### Two *Tenascin* Genes, *Tenascin-C* and *Tenascin-W*, Play Important Roles in myocardial migration in embryos raised at low temperature

**Figure 6:**
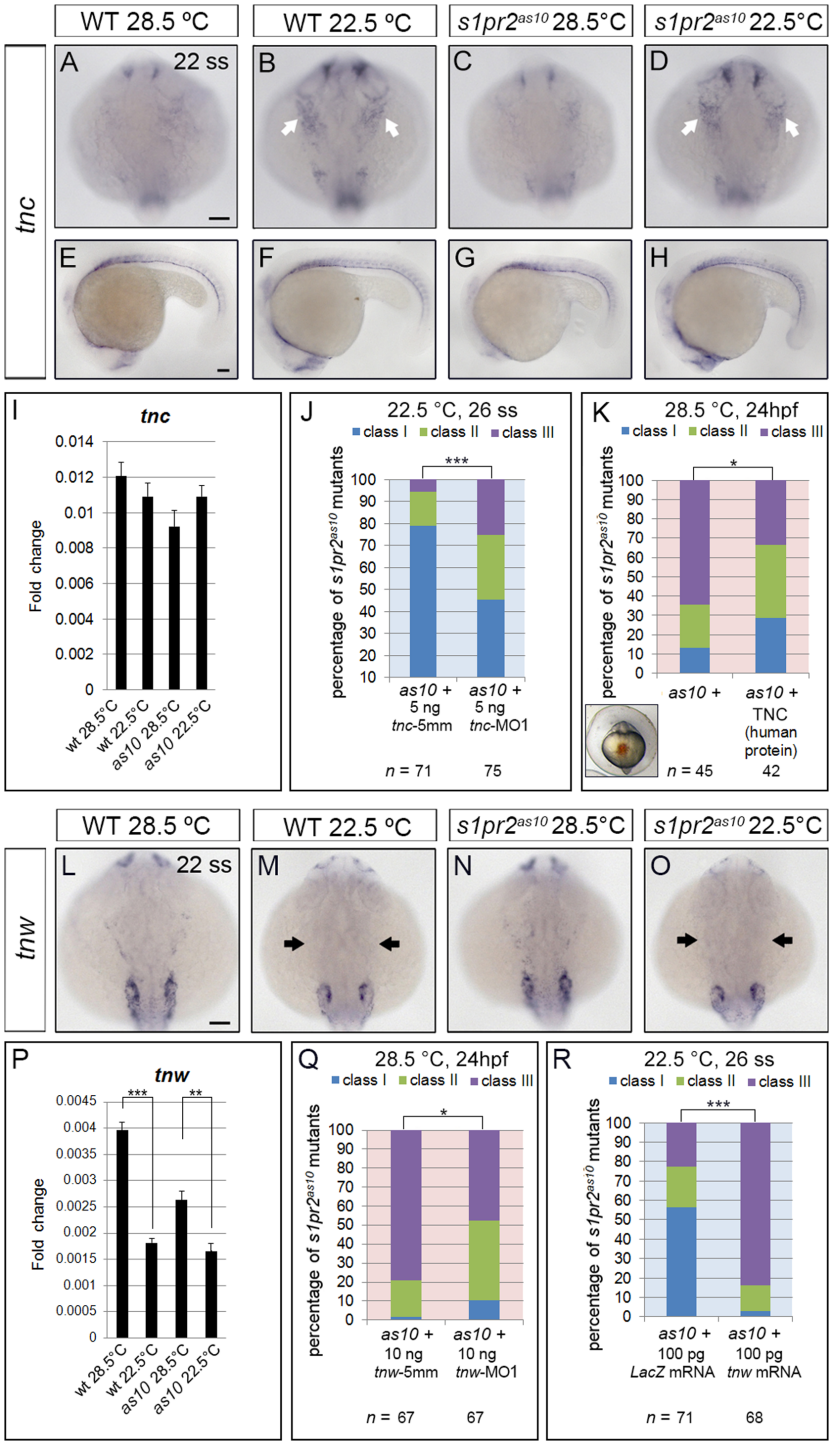
Expression of two *tenascin* genes is affected by temperature. *In situ* hybridization against *tenascin-c* (*tnc*) (A-H) and *tenascin-w* (*tnw*) (L-O) in 22-ss wild-type (WT) and *s1pr2^as10^* (*as10*) mutant embryos raised at 28.5°C or 22.5°C. Expression of *tnc* increased in the pharyngeal arches (white arrows in B and D) and the cells between the brain and eyes of embryos raised at 22.5°C, but was unaffected in the trunk somite region (E-H). (I) qRT-PCR revealed a trend towards increased expression of *tnc* mRNA in *s1pr2^as10^* mutant embryos raised at 22.5°C. (J) Knockdown of *tnc* expression with *tnc*-MO1 in *s1pr2^as10^* mutants increased the percentage of 26-ss embryos raised at 22.5°C with the Class III cardia bifida phenotype. (K) Injection of *s1pr2^as10^* mutant embryos raised at 28.5°C with human TNC protein partially rescued cardia bifida phenotypes at 24 hpf. (L-O) Decreased *tnw* expression in all tissues, including scattered epidermal cells in the head (black arrows in M and O) was observed in both 22-ss WT and *s1pr2^as10^* mutants raised at 22.5°C. (P) qRT-PCR was used to confirm that expression of *tnw* mRNA is significantly reduced in 22-ss WT and *s1pr2^as10^* mutant embryos raised at 22.5°C. (Q) Knockdown of *tnw* with *tnw*-MO1 in *s1pr2^as10^* mutant embryos raised at 28.5°C partially rescued cardia bifida phenotypes at 24 hpf. (R) Injection of *s1pr2^as10^* mutant embryos raised at 22.5°C with 100 pg *tnw* mRNA increased the percentage of 26-ss embryos with the Class III cardia bifida phenotype. Scale bars  = 100 µm. The error bars indicate the standard error. Statistical significance was determined using Student’s *t*-test. * indicates *p*<0.05, ** indicates *p*<0.01, *** indicates *p*<0.001.

In addition to *fn1*, expression levels of two other extracellular matrix genes, *tenascin-C* (*tnc*) and *tenascin-W* (*tnw*), were affected by incubation at low temperature. The *tnc* gene can produce four different isoforms through differential splicing. By using RNA probes against exons common to all *tnc* isoforms for *in situ* hybridization, we detected increased expression of *tnc* at the pharyngeal arches and in the cells between the brain and eyes of 22-ss WT and *s1pr2^as10^* mutant embryos raised at 22.5°C as compared to those raised at 28.5°C (Figure 6B and 6D vs. 6A and 6C). However, expression of *tnc* in the somite region was not affected by the temperature of incubation (Figure 6E–6H). Accordingly, the use of qRT-PCR to measure *tnc* mRNA did not uncover significant differences between WT and *s1pr2^as10^* mutant embryos raised at 22.5°C as compared to those raised at 28.5°C (Figure 6I). We subsequently used a previously published *tnc*-MO1 that can prevent the translation of all *tnc* isoforms [22] and designed a *tnc*−5mm MO based on the *tnc*-MO1 sequence for use as controls. Additionally, we used the previously published *tnc*-MO2, which blocks splicing of *tnc* exon 1 [22]. Mutant *s1pr2^as10^* embryos incubated at 22.5°C and injected with either *tnc*-MO1 or *tnc*-MO2 presented with more severe cardia bifida phenotypes, as compared to *tnc*−5 mm injected embryos (Figure 6J and Figure S9A). We also injected human TNC protein into the midline region of *s1pr2^as10^* mutants, and found the percentage of embryos with class III severe cardia bifida defects was reduced from 64% to 33% (Figure 6K). These results indicate that low temperature rescues *s1pr2^as10^* cardiac phenotype partially due to increased expression of *tnc*.

DNA microarray analysis also revealed decreased expression of another *tenascin* gene, *tnw,* in 22-ss WT and *s1pr2^as10^* mutant embryos raised at 22.5°C. Whole-mount *in situ* hybridization and qRT-PCR confirmed that *tnw* mRNA expression was reduced in all tissues, including the LPM, of both 22-ss WT and *s1pr2^as10^* mutant embryos raised at 22.5°C (Figure 6M, 6O and 6P). We subsequently designed morpholinos (*tnw*-MO1 and *tnw*-MO2) against the 5′ untranslated region (UTR) of *tnw* to prevent its translation; we also designed a *tnw*−5 mm based on the *tnw*-MO1 sequence as a control. In order to test the efficiency and specificity of *tnw*-MOs, we fused *EGFP* in frame with the *tnw* 5′UTR under the control of the CMV enhancer/promoter, and co-injected this construct with *tnw*-MO1, *tnw*-MO2, or *tnw*−5 mm into one-cell zygotes. As shown in Figure S9D and E, we detected EGFP expression in the majority of embryos co-injected with *tnw*−5 mm and *CMV*-*tnwUTR*-*EGFP* plasmid. In contrast, the majority of embryos (85%) co-injected with *tnw*-MO1 and *CMV*-*tnwUTR*-*EGFP* did not express GFP. However, 30% of embryos co-injected with *tnw*-MO2 and *CMV*-*tnwUTR*-*EGFP* expressed GFP, indicating that *tnw*-MO1 is more efficient at blocking EGFP translation. This may be a consequence of *tnw*-MO2 targeting a region further upstream from that of *tnw*-MO1.

We then injected either *tnw*-MO1 or *tnw*-MO2 into *s1pr2^as10^* mutants raised at 28.5°C, and observed partial rescue of the cardia bifida phenotype at 24 hpf (Figure 6Q and Figure S9B). Injection of *tnw*-MO1 resulted in greater rescue of the cardia bifida phenotype than with *tnw*-MO2; this may be attributed to the reduced efficiency of *tnw*-MO2 in blocking translation. Furthermore, some of the rescued embryos contained a single heart tube and displayed normal circulation (data not shown). Interestingly, injection of *tnw* mRNA at the one-cell stage caused a highly severe cardia bifida phenotype in *s1pr2^as10^* mutants, which was observed even at 22.5°C (Figure 6R).

We also injected *fn1*-MO, *tnc*-MO1, or *tnw* mRNA into wild type embryos to investigate their effects on myocardial migration. We found that the percentage of *tnc*-MO1-injected morphants (26%) which developed a horseshoe-shaped heart at 22ss was similar to that of control embryos (27%) (Figure S10A and S10B). The percentage of *tnw* mRNA-injected embryos with a horseshoe-shaped heart at 22ss was greater (38%) than that of control, and 12% of these embryos also exhibited cardia bifida (Figure S10D). The percentage of embryos with horseshoe-shaped hearts at 22 ss was increased further by *fn1*-MO1-injection (69%), and 9% of these embryos exhibited cardia bifida (9%) (Figure S10C); these results indicate that fibronectin plays a more important role in regulating myocardial migration than tenascin-C or tenascin-W. However, the majority (93%) of embryos injected with a mixture of *tnc*-MO1, *fn1*-MO, and *tnw* mRNA exhibited cardia bifida at 22 ss (Figure S10E); this result further demonstrates that tenascin-C and fibronectin positively regulate, while tenascin-W negatively regulates, myocardial migration in zebrafish embryos.

Together, these data indicate that *tnc* and *tnw* play opposing roles in zebrafish myocardial migration, and that low temperature mitigates cardia bifida by altering the expression of ECM genes: *fn1* and *tnc* are up-regulated, and *tnw* is down-regulated.

### Reactive Oxygen Species (ROS) Mediate Mitigation of Cardia Bifida by Low Temperature

**Figure 7:**
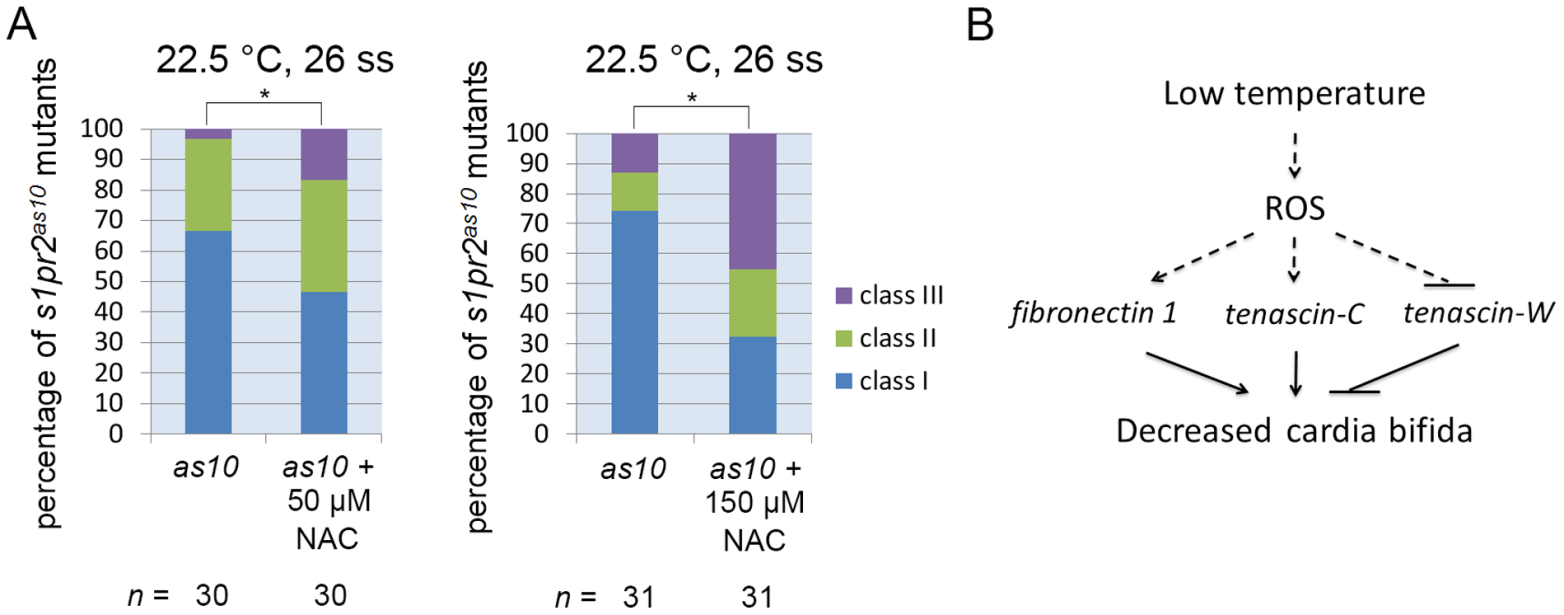
Reactive oxygen species (ROS) mediate mitigation of cardia bifida in zebrafish embryos incubated at low temperature. (A) *s1pr2^as10^* mutant embryos raised at 22.5°C were treated with 50 or 150 µM N-acetyl cysteine (NAC) at the tailbud stage, and were then incubated at 22.5°C. Different degrees of myocardial migration defects were observed at the 26 ss. Class I (single heart tube), Class II (cardiomyocytes in close proximity but not in contact), and Class III (two separate hearts). (B) A proposed model showing how low temperature mitigates cardia bifida in zebrafish embryos. Statistical significance was determined using Student’s *t*-test. * indicates *p*<0.05.

Cold exposure was previously shown to increase expression of peroxisome proliferator-activated receptors (PPARs) and uncoupling proteins (UCPs) in the zebrafish brain, to prevent oxidative damage and to maintain metabolic balance and homeostasis [31]. We investigated whether ROS mediates mitigation of the cardia bifida phenotype in *s1pr2^as10^* mutants by low temperature treatment. To test this hypothesis, we treated *s1pr2^as10^* mutant embryos with either 50 or 150 µM of N-acetyl cysteine (NAC), a ROS scavenger, at the tailbud stage, and incubated the embryos at 22.5°C. As shown in Figure 7, we detected a dose-dependent of NAC on the percentage of *s1pr2^as10^* mutant embryos with a class I single heart tube at 26 ss when incubated at low temperature; class I heart tubes were reduced by NAC to 46–32% as compared to 67–73% in untreated *s1pr2^as10^* mutant embryos. This result suggests that ROS may mediate mitigation of cardia bifida by low temperature.

## Discussion

Previous reports have demonstrated that changes in temperature affect various aspects of development and gene expression [29], [30]. In this study, we have demonstrated that temperature also affects the timing of cardiac cone formation. We have specifically demonstrated that low temperature (22.5°C) mitigates cardia bifida in both *s1pr2^as10^* mutant and *gata5* and *bon* morphant embryos. DNA microarray and digital gene expression analyses revealed that low temperature regulates expression of several extracellular matrix (ECM) genes to control myocardial migration.

An increase in *fn1* expression at the midline region and bilateral anterior lateral plate mesoderm (LPM) was identified in both 22-ss WT and *s1pr2^as10^* mutant embryos raised at 22.5°C (Figure 5B, and 5D and Figure S8). Injection of *s1pr2^as10^* mutant embryos with *fn1*-MO abolished the rescue of cardia bifida by low temperature (Figure 5J), while injection with human fibronectin protein decreased the percentage of *s1pr2^as10^* mutant embryos raised at 28.5°C with class III severe cardia bifica at 24 hpf (Figure 5K). During myocardial precursor migration, fibronectin secreted by the endoderm and/or endocardial precursors is deposited at the midline and laterally around the myocardial precursors, to maintain the integrity of myocardial epithelia, and mediate interactions between the endoderm and migrating myocardial precursors [8], [15]. It has been demonstrated that knockdown of *mil*/*edg5* results in cardia bifida and decreased levels of fibronectin [11]. Furthermore, injection of the midline with fibronectin partially rescued the cardia bifida phenotype in *mil* mutant embryos [15]. All of these findings suggest that both *mil*/*edg5* and low temperature play a positive role in regulating myocardial migration. Proper deposition of extracellular matrix proteins is also important for normal cardiac function after formation of the four-chambered heart, and excess collagen and fibronectin deposition results in cardiac fibrosis in cardiac hypertrophy. Reactive oxygen species (ROS) have been shown to up-regulate expression of profibrotic genes, such as *col2a1*, *col1a1*, and *fn1*, thereby resulting in direct extracellular matrix deposition in a transgenic mouse model, and resulting in cardiac hypertrophy [32].

Tenascins are a family of large multimeric extracellular matrix glycoproteins involved in cell adhesion, migration and proliferation [33], [34]. Tenascin-C has been shown to either promote or inhibit cell adhesion and migration, depending on the cell type. Addition of tenascin-C stimulated the migration of human adult dermal fibroblasts cultured within matrices comprised of fibronectin [35]. Smooth muscle cells cultured on tenascin-C substrate exhibited increased expression of αv-integrin and elevated phosphorylation of focal adhesion kinase (FAK), followed by enhanced migration [36]. Tenascin-C also promoted bovine retinal endothelial cell migration by enhancement of FAK phosphorylation [37]. On the other hand, high levels of tenascin-C produced by glioma tumour tissues actively hindered T-cell migration, resulting in an accumulation of T cells in the peritumoral region [38]. Although the function of tenascin-W and its gene regulation has not been well-characterized, it has been shown to confer anti-adhesive properties [39]. Addition of soluble tenascin-W was reported to inhibit the formation of lamellipodia with stress fibers and focal adhesion complexes in a murine myoblast cell line (C2C12) cultured on fibronectin. Various factors, including inflammatory cytokines, growth factors, oxidative stress, and hypoxia, have been shown to induce *tnc* expression [40]. In neonatal rat cardiomyocytes, mechanical strain increased *tnc* expression by activating nuclear factor-κB through ROS [41]. Here, we observed up-regulation of *tnc* and down-regulation of *tnw* in embryos raised at low temperature (Figures 5 and 6). MO knockdown and mRNA or protein rescue confirmed the opposing roles of tenascin-C and tenascin-W in mitigating cardia bifida in zebrafish embryos. Moreover, we found that the addition of N-acetyl cysteine (NAC), a ROS scavenger, decreased mitigation of cardia bifida in *s1pr2^as10^* mutant embryos raised at 22.5°C (Figure 7A).

Based on our results and previously published studies, we propose a model describing how low temperature mitigates cardia bifida in zebrafish embryos (Figure 7B). Low temperature may enhance ROS production in cranial neural crest cells, endocardial and myocardial cells, and endoderm (in a similar manner to ROS induction in zebrafish brain upon cold exposure [31]), as these tissues are more vulnerable to oxidative stress [42]–[47]. The presence of ROS in the endoderm and anterior LPM may then up-regulate *fn1* expression, resulting in the secretion of fibronectin in the midline and bilateral anterior LPM (Figure 5 and Figure S8). Meanwhile, ROS production in the cranial neural crest derived-bilateral pharyngeal arches may activate *tnc* expression, thereby promoting secretion of tenascin-C near migrating bilateral cardiomyocytes (Figure 6). Increased levels of secreted tenascin-C may enhance integrin expression and promote FAK phosphorylation in migrating bilateral cardiomyocytes, thereby facilitating their interaction with fibronectin, formation of focal adhesion complexes, actin polymerization, and protrusion [37], [48]–[50]. Increased fibronectin around migrating cardiomyocytes can further promote the integrity of myocardial epithelia, which is a prerequisite of myocardial migration [8]. Since tenascin-W is known to inhibit cell adhesion to fibronectin, ROS may also down-regulate *tnw* expression, consequently preventing secretion of tenascin-W from scattered epidermal cells in the head region, and thereby allowing migrating cardiomyocytes to interact with fibronectin [51]. Furthermore, a combinatorial effect of up-regulation of *tnc* and *fn1*, and down-regulation of *tnw* are required to mitigate cardia bifida of *s1pr2^as10^* mutant embryos and *gata5* and *bon* cardia bifida morphants upon low temperature treatment.

In conclusion, our identification of the *s1pr2^as10^* mutation serendipitously led to our finding that incubation at low temperature mitigates cardia bifida, a phenomena caused by up-regulation of *fn1* and *tnc* and down-regulation of *tnw*. In addition, ROS mediates mitigation of cardia bifida upon low temperature treatment. Future studies may be expected to delineate the mechanisms underlying regulation of *fn1*, *tnc* and *tnw* expression by ROS under low temperatures.

## Supporting Information

Figure S1 **Genetic mapping of the** ***s1pr2^as10^*** **mutant gene.** (A) Meiotic map of the *s1pr2^as10^* locus. The numbers above the line indicate the number of meiotic recombination events out of the total number of meioses. (B) An enlarged view of the *mil*/*edg5* locus shows the insertion in intron 1. Three primers (F1, R1, and I1) were used to confirm the insertion. (C) A 182-bp DNA fragment amplified using primers F1 and R1 was detected in wild-type (WT) and *s1pr2^as10^* heterozygous mutants, whereas a 426*-*bp DNA fragment amplified using primers I1 and R1 was detected in *s1pr2^as10^* heterozygous and homozygous mutant embryos. (D) Expression of *mil/edg5* mRNA was reduced in *s1pr2^as10^* mutants prior to 96 hpf, as revealed by qRT-PCR. WT (+, W); *s1pr2^as10^* mutant (−, M). The error bars indicate the standard error.
(TIF)

Figure S2 **Dosage curve of an** ***edg5*** **-morpholino antisense oligomer (MO) and its association with cardia bifida.** (A) *Tg*(*cmlc2:EGFP, cmlc2:H2AFZmCherry*)*^cy13^* embryos injected with different doses of *edg5*-MO and *edg5*-5 mm exhibited varying degrees of myocardial migration defects, ranging from Class I (a single heart tube) to Class II (cardiomyocytes either in close proximity or in contact) and Class III (separated cardiomyocytes) at 24 hpf. Scale bar = 100 µm. (B) Percentages of Class I, II and III myocardial migration defects in *Tg*(*cmlc2:EGFP, cmlc2:H2AFZmCherry*)*^cy13^* embryos injected with 1–16 ng of *edg5*-MO at 24 hpf.
(TIF)

Figure S3 **Injection of** ***s1pr2^as10^*** **mutants with** ***mil*** **/** ***edg5*** **mRNA partially rescued the cardia bifida phenotype.** Injection of *s1pr2^as10^* mutant embryos with *mil*/*edg5* mRNA, but not *LacZ* mRNA, decreased the percentage of embryos with the Class III cardia bifida phenotype. Statistical significance was determined using Student’s *t*-test. * indicates *p*<0.05.
(TIF)

Figure S4 **The 16 ss to the 22 ss is the critical time interval for rescue of the** ***s1pr2^as10^*** **mutant heart at 22.5°C.** *s1pr2^as10^* mutants raised at 28.5**°**C (red line) or 22.5**°**C (blue line) at different stages were rescued to differing levels, as determined by the percentage of embryos containing one atrium and one ventricle at the protruding-mouth stage.
(TIF)

Figure S5 **Low temperature treatment cannot rescue the cardia bifida phenotype of** ***mil*** **mutants and 20 ng** ***gata5*** **MO-injected embryos.** (A) *mil^m93^* mutant embryos were incubated at 28.5°C or 22.5°C. Different degrees of myocardial migration defects were observed from prim 5-prim 15. (B) Percentages of each class of myocardial migration defect in 20 ng *gata5*-MO or *bon*-MO-injected embryos raised at 28.5 or 22.5°C at the 26 ss. Myocardial migration defects were observed at prim-5. Class I (single heart tube), Class II (cardiomyocytes in close proximity but not in contact), and Class III (two separate hearts).
(TIF)

Figure S6 **Anterior gut migration defects in** ***gata5*** **morphants can be rescued by low temperature treatment.** Embryos injected with 10 ng *gata5* MO were incubated at 28.5°C (A) or 22.5°C (B). Embryos were harvested at prim-25 and stained with *foxa1* RNA probe. (C). The distance between two lateral anterior gut tubes was significantly different between morphants incubated at 28.5°C or 22.5°C. Scale bars  =  100 μm. The error bars indicate the standard error. Statistical significance was determined using Student’s *t*-test. *** indicates *p* <0.001.
(TIF)

Figure S7 ***mil/edg5*** **levels were similar in embryos raised at 28.5°C or 22.5°C.** qRT-PCR measurement of *mil*/*edg5* levels in 22-ss WT or *s1pr2^as10^* mutant embryos raised at 28.5 or 22.5°C. The error bars indicate the standard error.
(TIF)

Figure S8 **Low temperature increases fibronectin 1 expression in the midline region.** Immunohistochemistry was used to demonstrate increased fibronectin 1 expression at the midline region (white arrows) of 22-ss wild type (WT) and *s1pr2^as10^* mutant embryos raised at 22.5°C. Scale bars  = 100 µm.
(TIF)

Figure S9 **Evaluation of the roles of** ***tnc*** **and** ***tnw*** **in the mitigation of cardia bifida, and of the efficiency and specificity of** ***tnw*** **MOs.** (A) Knockdown of *tnc* with *tnc*-MO2 in *s1pr2^as10^* mutants increased the percentages of 26-ss embryos raised at 22.5°C with the Class II and Class III cardia bifida phenotype. (B) Knockdown of *tnw* with *tnw*-MO2 in *s1pr2^as10^* mutant embryos raised at 28.5°C partially rescued cardia bifida phenotypes at 24 hpf. Class I (a single heart tube) to Class II (cardiomyocytes either in close proximity or in contact) and Class III (separated cardiomyocytes). Statistical significance was determined using Student’s *t*-test. * indicates *p* <0.05, ** indicates *p* <0.01. (C) Diagram indicating the relative binding positions of two *tnw*-MOs in the 5’untranslated region of *tnw* mRNA. (D) Green fluorescence can be detected in embryos co-injected with *CMV-tnwUTR-EGFP* and *tnw*-5mm at 24 hpf. Green fluorescence was not observed in the majority of embryos co-injected with *CMV-tnwUTR-EGFP* and *tnw*-MO1, while green fluorescence was detected in 30% of embryos co-injected with *CMV-tnwUTR-EGFP* and *tnw*-MO2. (E) Percentage of embryos expressing EGFP following co-injection of *CMV-tnwUTR-EGFP* with *tnw*-5mmMO, *tnw*-MO1 or *tnw*-MO2. The error bars indicate the standard error.
(TIF)

Figure S10 **Knockdown of** ***fn1*** **or** ***tnc*** **and overexpression of** ***tnw*** **results in cardia bifida in wild type embryos.** Wild type embryos at the one-cell zygote stage were injected with 5 ng *tnc*-MO1 (B), 2.5 ng *fn1*-MO (C), 100 pg *tnw* mRNA (D), or a mixture of *tnc*-MO1, *fn1*-MO, and *tnw* mRNA (E), and incubated at 28.5°C. Un-injected embryos were used as a control (A). Embryos were harvested at the 22 ss and stained with *cmlc2*.
(TIF)

Table S1 **Circulation defect of** ***s1pr2^as10^*** **mutant embryos from different genotypes could be partially rescued when raised at 22.5°C.** The statistics of the offspring derived from different genotypes of *s1pr2^as10^* mutant raised at 28.5°C or 22.5°C. Genotype labeled with +/− or −/− indicated the heterozygous or homozygous *s1pr2^as10^* mutants respectively. For example, among 213 embryos from heterozygous mutants intercross (+/− x +/−) raised at 28.5°C, 162 embryos showed normal wild-type phenotype (a), 46 embryos contained tail blisters phenotype and established no blood circulation (b), and 5 embryos contained tail blisters with normal circulation (c). Mendel ratio was calculated by number of embryos with tail blister phenotype divided by number of total embryos. Rescue of tail blister with circulation phenotype was observed in offspring derived from different genotypes of *s1pr2^as10^* mutant raised at 22.5°C. Rescue percentage of tail blister with circulation phenotype was calculated by number of embryos showing tail blister with circulation phenotype divided by total number of embryos showing tail blister phenotype.
(DOC)

Table S2 **Up- and down-regulated genes in** ***s1pr2^as10^*** **mutant at 22.5°C as determined by digital gene expression (DGE) analysis.** Double-stranded cDNA from 22 ss-*s1pr2^as10^* mutant embryos raised at 28.5°C and 22.5°C were synthesized for next generation sequencing. The Up- and down-regulated genes at 22.5°C were subsequently analyzed by pathway analysis according to Kyoto Encyclopedia of Genes and Genomes (KEGG) database. Several groups of genes were selected for further analysis.
(DOC)
