# Supplementary figures and images for: Low Temperature Mitigates Cardia Bifida in Zebrafish Embryos
Source: PLoS One. 2013 Jul 26;8(7):e69788. doi: 10.1371/journal.pone.0069788 (PMC3724881; doi:10.1371/journal.pone.0069788)

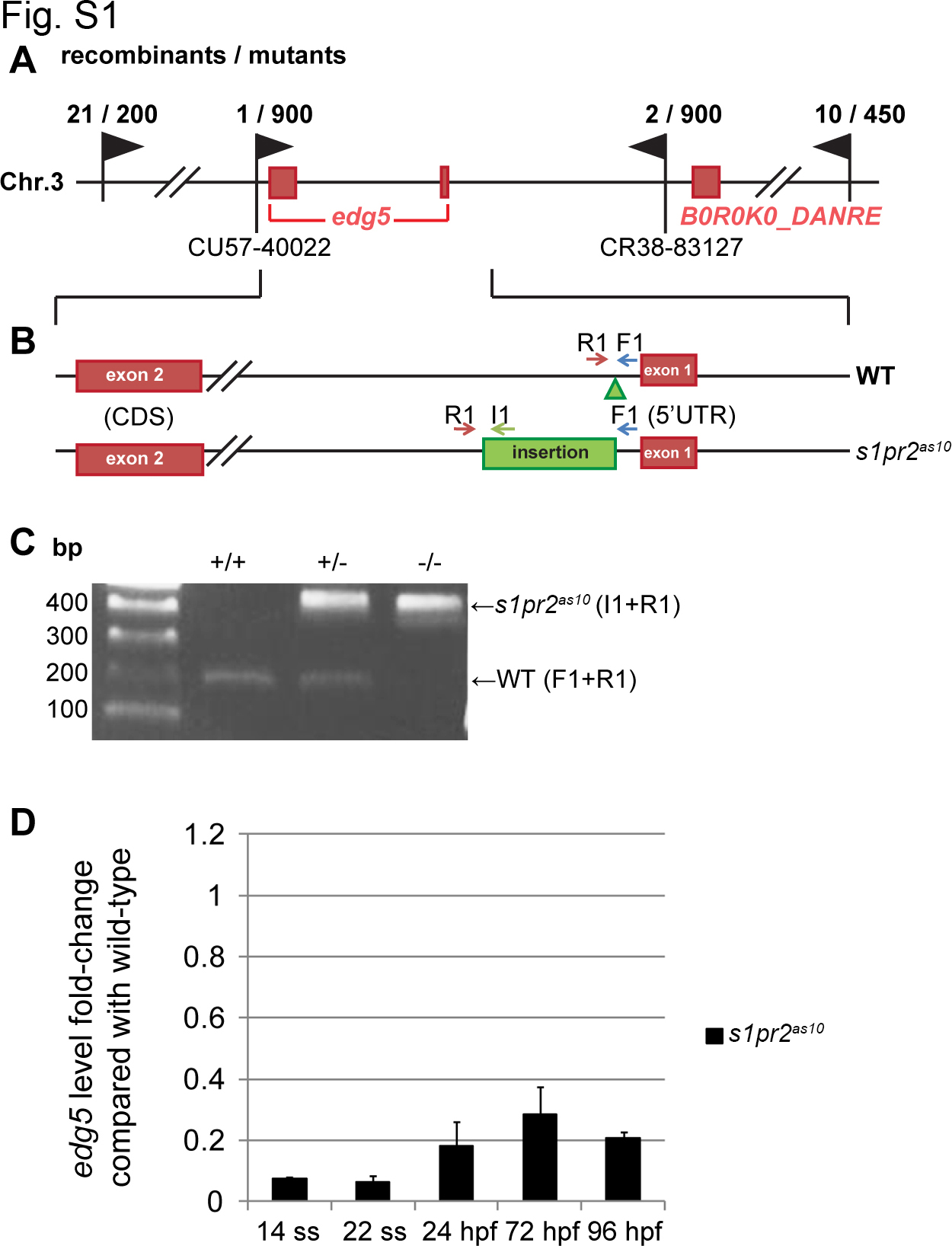

Supplement: Figure S1 — Genetic mapping of the s1pr2as10 mutant gene. (A) Meiotic map of the s1pr2as10 locus. The numbers above the line indicate the number of meiotic recombination events out of the total number of meioses. (B) An enlarged view of the mil/edg5 locus shows the insertion in intron 1. Three primers (F1, R1, and I1) were used to confirm the insertion. (C) A 182-bp DNA fragment amplified using primers F1 and R1 was detected in wild-type (WT) and s1pr2as10 heterozygous mutants, whereas a 426-bp DNA fragment amplified using primers I1 and R1 was detected in s1pr2as10 heterozygous and homozygous mutant embryos. (D) Expression of mil/edg5 mRNA was reduced in s1pr2as10 mutants prior to 96 hpf, as revealed by qRT-PCR. WT (+, W); s1pr2as10 mutant (−, M). The error bars indicate the standard error. (TIF) [file pone.0069788.s001.tif]

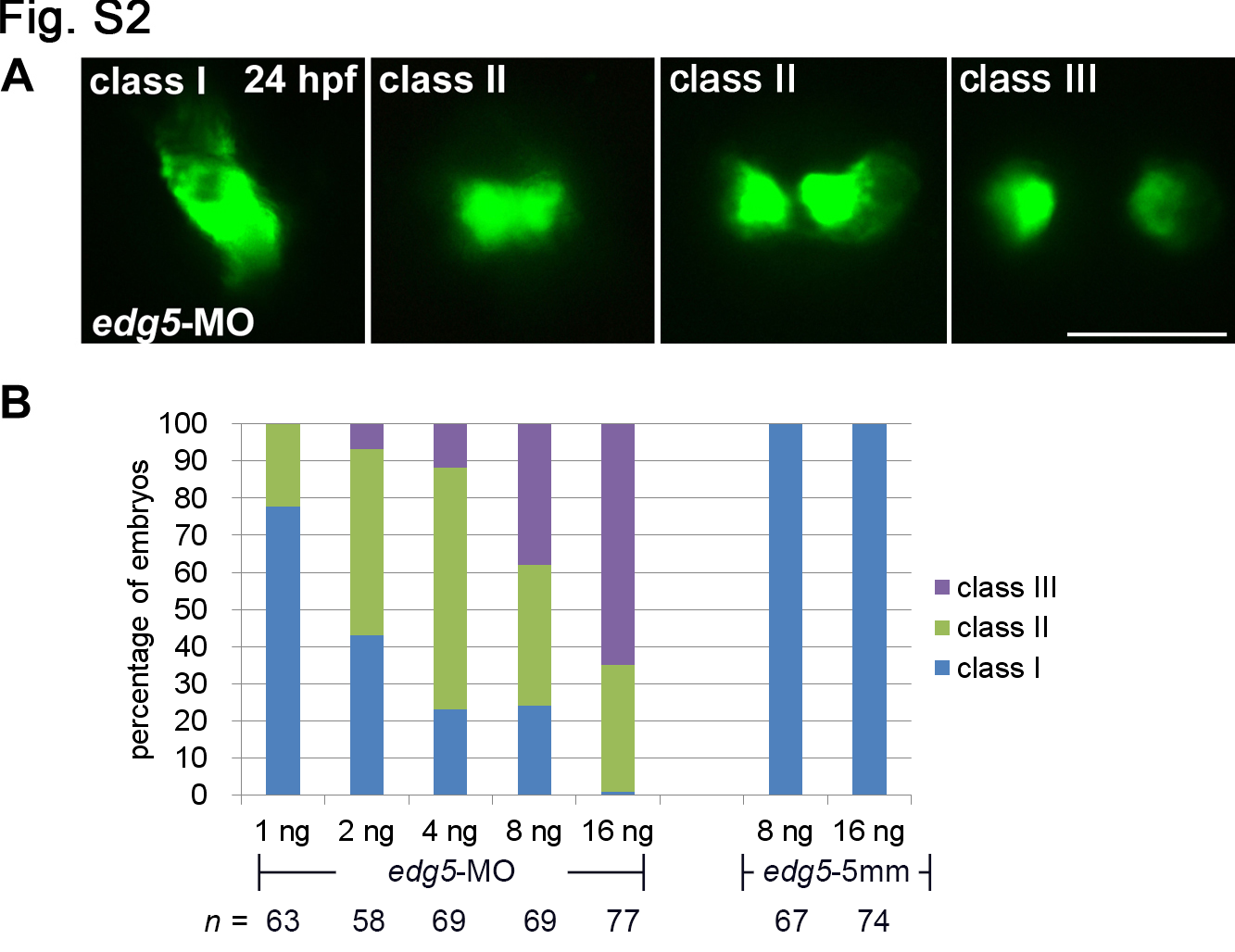

Supplement: Figure S2 — Dosage curve of an edg5 -morpholino antisense oligomer (MO) and its association with cardia bifida. (A) Tg(cmlc2:EGFP, cmlc2:H2AFZmCherry)cy13 embryos injected with different doses of edg5-MO and edg5-5 mm exhibited varying degrees of myocardial migration defects, ranging from Class I (a single heart tube) to Class II (cardiomyocytes either in close proximity or in contact) and Class III (separated cardiomyocytes) at 24 hpf. Scale bar = 100 µm. (B) Percentages of Class I, II and III myocardial migration defects in Tg(cmlc2:EGFP, cmlc2:H2AFZmCherry)cy13 embryos injected with 1–16 ng of edg5-MO at 24 hpf. (TIF) [file pone.0069788.s002.tif]

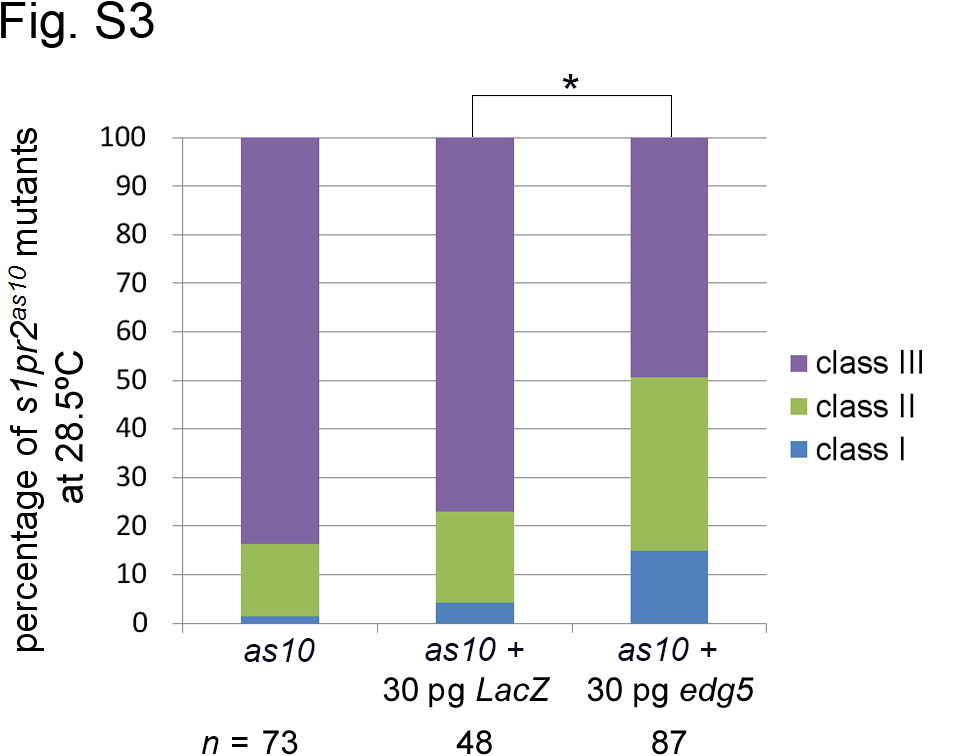

Supplement: Figure S3 — Injection of s1pr2as10 mutants with mil / edg5 mRNA partially rescued the cardia bifida phenotype. Injection of s1pr2as10 mutant embryos with mil/edg5 mRNA, but not LacZ mRNA, decreased the percentage of embryos with the Class III cardia bifida phenotype. Statistical significance was determined using Student’s t-test. * indicates p<0.05. (TIF) [file pone.0069788.s003.tif]

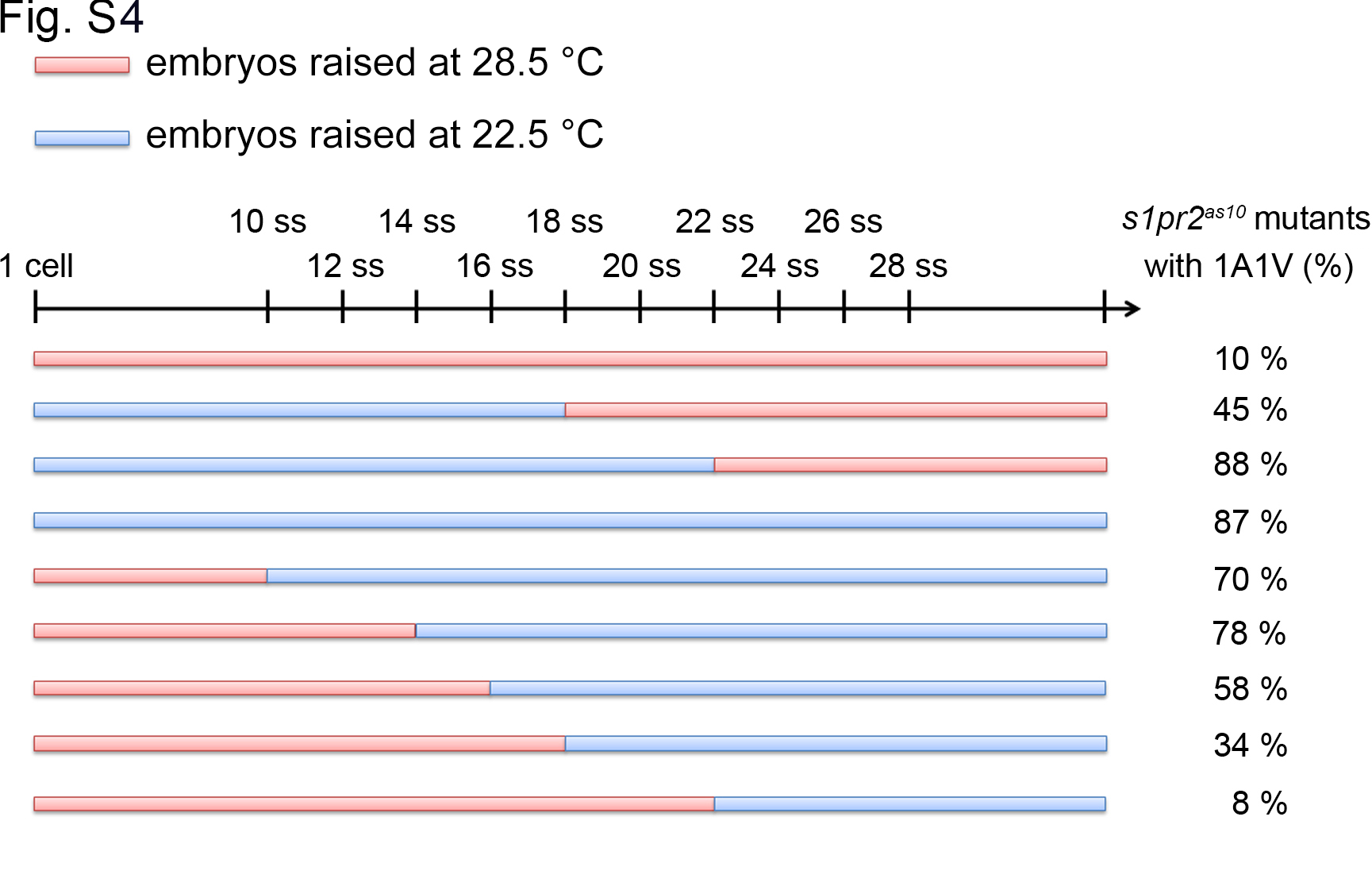

Supplement: Figure S4 — The 16 ss to the 22 ss is the critical time interval for rescue of the s1pr2as10 mutant heart at 22.5°C. s1pr2as10 mutants raised at 28.5°C (red line) or 22.5°C (blue line) at different stages were rescued to differing levels, as determined by the percentage of embryos containing one atrium and one ventricle at the protruding-mouth stage. (TIF) [file pone.0069788.s004.tif]

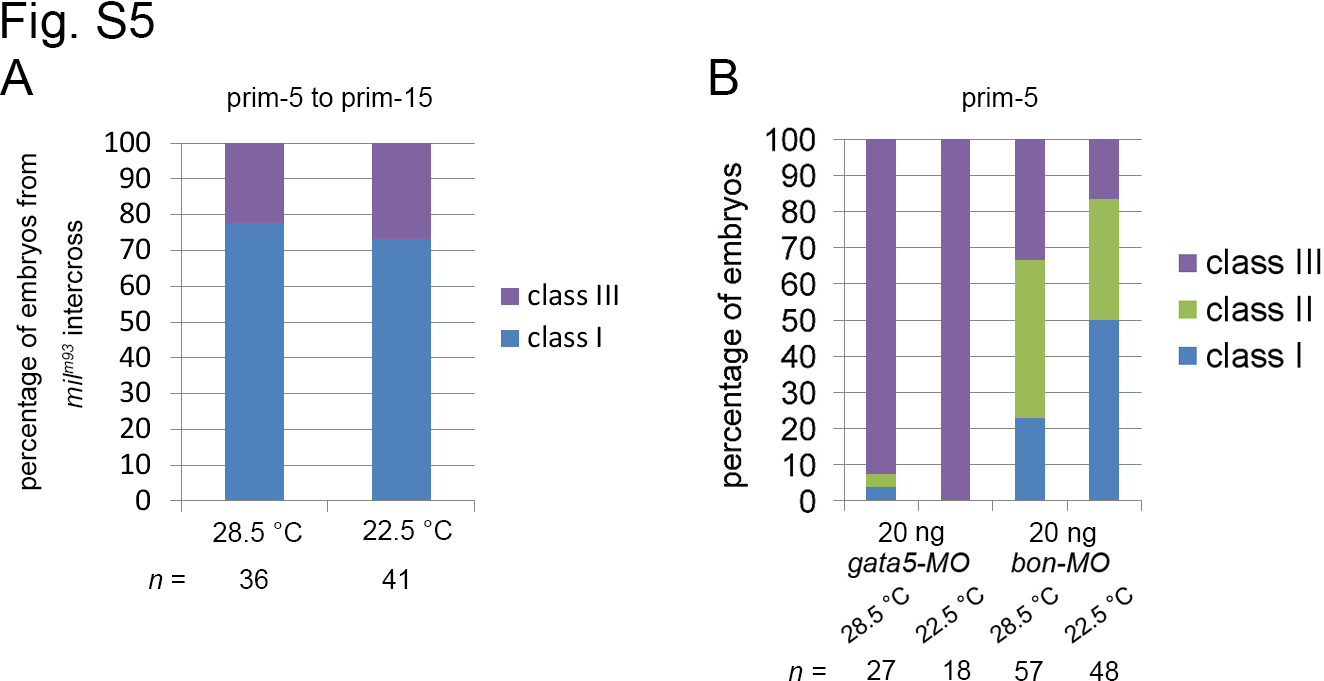

Supplement: Figure S5 — Low temperature treatment cannot rescue the cardia bifida phenotype of mil mutants and 20 ng gata5 MO-injected embryos. (A) milm93 mutant embryos were incubated at 28.5°C or 22.5°C. Different degrees of myocardial migration defects were observed from prim 5-prim 15. (B) Percentages of each class of myocardial migration defect in 20 ng gata5-MO or bon-MO-injected embryos raised at 28.5 or 22.5°C at the 26 ss. Myocardial migration defects were observed at prim-5. Class I (single heart tube), Class II (cardiomyocytes in close proximity but not in contact), and Class III (two separate hearts). (TIF) [file pone.0069788.s005.tif]

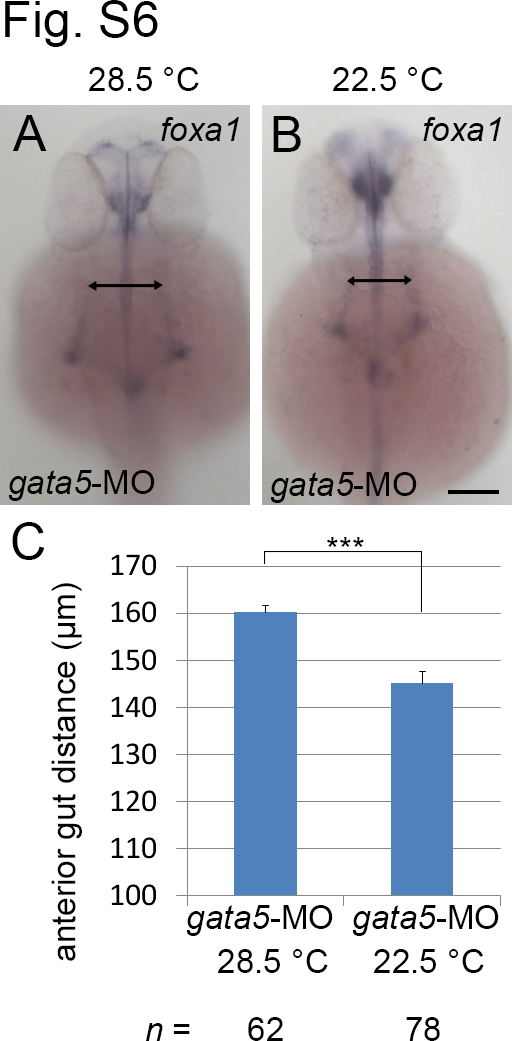

Supplement: Figure S6 — Anterior gut migration defects in gata5 morphants can be rescued by low temperature treatment. Embryos injected with 10 ng gata5 MO were incubated at 28.5°C (A) or 22.5°C (B). Embryos were harvested at prim-25 and stained with foxa1 RNA probe. (C). The distance between two lateral anterior gut tubes was significantly different between morphants incubated at 28.5°C or 22.5°C. Scale bars = 100 μm. The error bars indicate the standard error. Statistical significance was determined using Student’s t-test. *** indicates p <0.001. (TIF) [file pone.0069788.s006.tif]

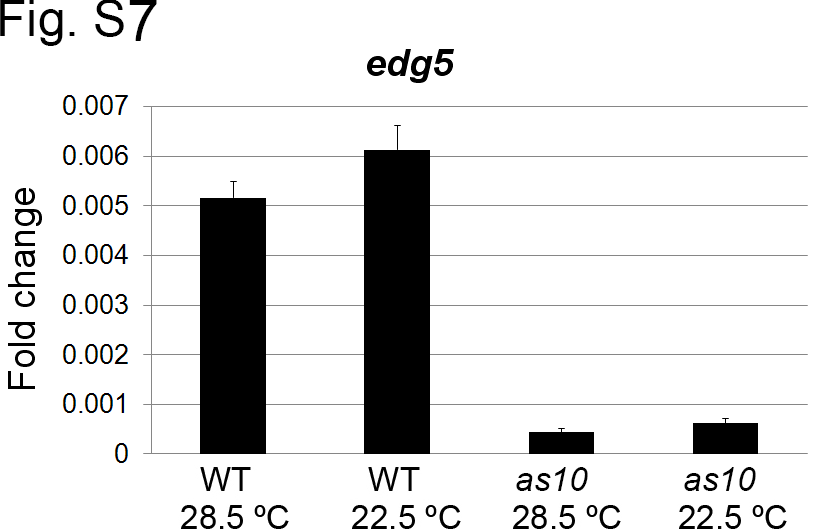

Supplement: Figure S7 — mil/edg5 levels were similar in embryos raised at 28.5°C or 22.5°C. qRT-PCR measurement of mil/edg5 levels in 22-ss WT or s1pr2as10 mutant embryos raised at 28.5 or 22.5°C. The error bars indicate the standard error. (TIF) [file pone.0069788.s007.tif]

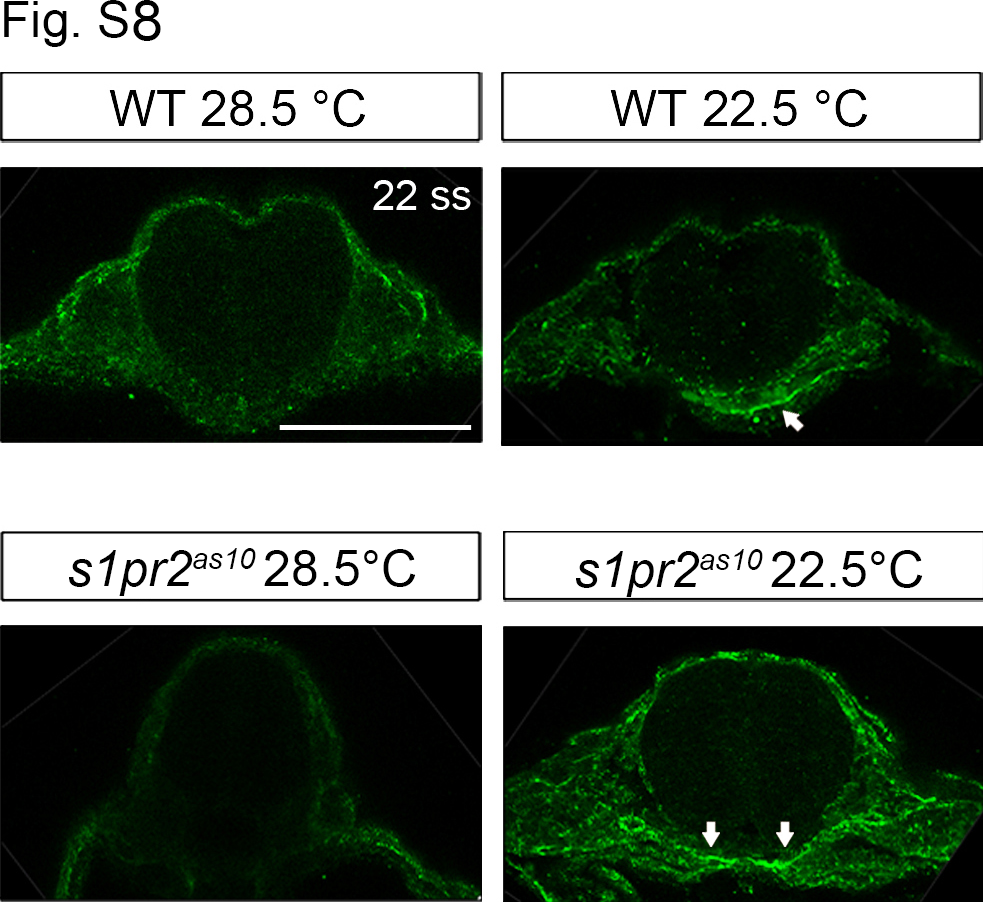

Supplement: Figure S8 — Low temperature increases fibronectin 1 expression in the midline region. Immunohistochemistry was used to demonstrate increased fibronectin 1 expression at the midline region (white arrows) of 22-ss wild type (WT) and s1pr2as10 mutant embryos raised at 22.5°C. Scale bars = 100 µm. (TIF) [file pone.0069788.s008.tif]

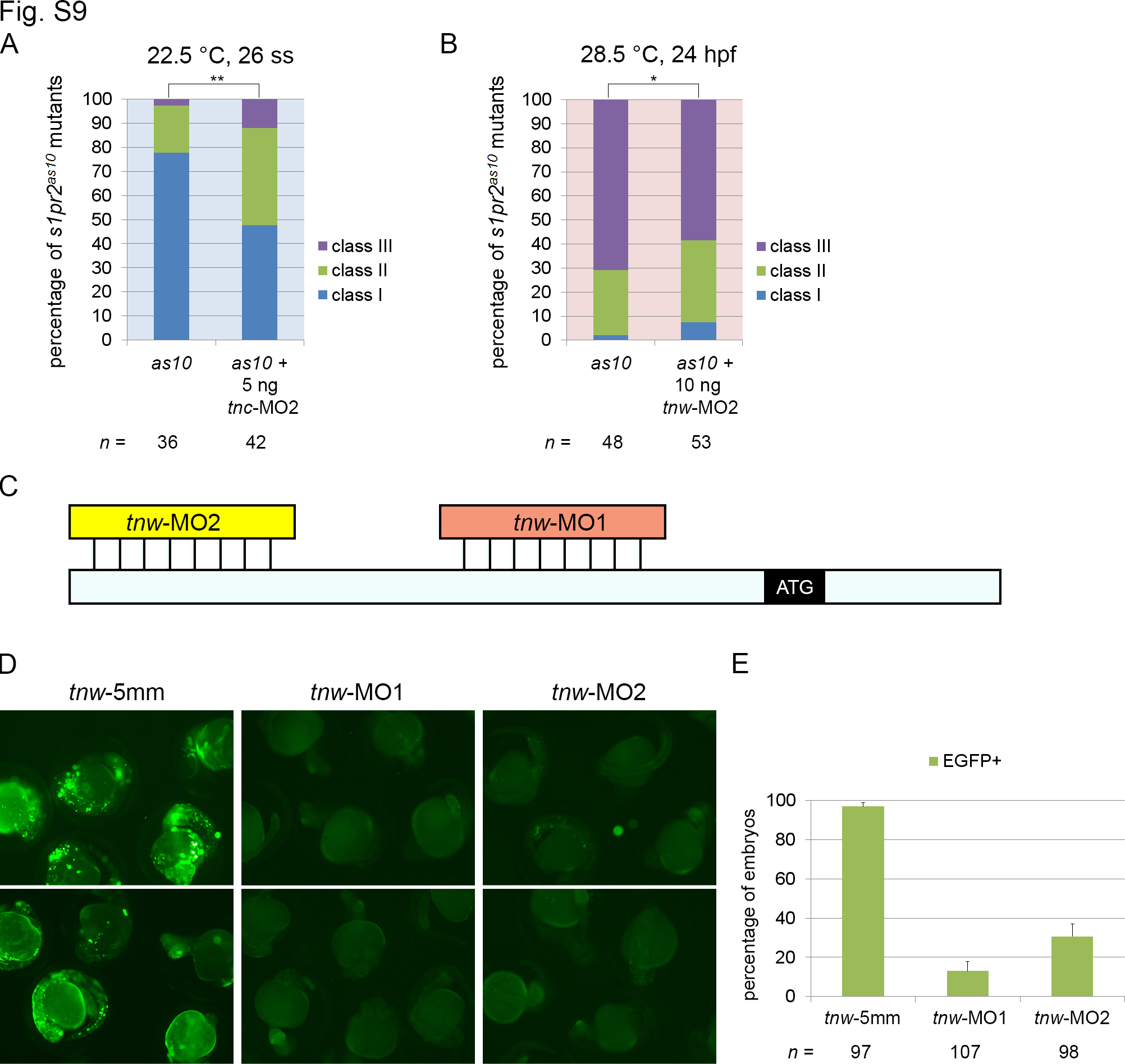

Supplement: Figure S9 — Evaluation of the roles of tnc and tnw in the mitigation of cardia bifida, and of the efficiency and specificity of tnw MOs. (A) Knockdown of tnc with tnc-MO2 in s1pr2as10 mutants increased the percentages of 26-ss embryos raised at 22.5°C with the Class II and Class III cardia bifida phenotype. (B) Knockdown of tnw with tnw-MO2 in s1pr2as10 mutant embryos raised at 28.5°C partially rescued cardia bifida phenotypes at 24 hpf. Class I (a single heart tube) to Class II (cardiomyocytes either in close proximity or in contact) and Class III (separated cardiomyocytes). Statistical significance was determined using Student’s t-test. * indicates p <0.05, ** indicates p <0.01. (C) Diagram indicating the relative binding positions of two tnw-MOs in the 5’untranslated region of tnw mRNA. (D) Green fluorescence can be detected in embryos co-injected with CMV-tnwUTR-EGFP and tnw-5mm at 24 hpf. Green fluorescence was not observed in the majority of embryos co-injected with CMV-tnwUTR-EGFP and tnw-MO1, while green fluorescence was detected in 30% of embryos co-injected with CMV-tnwUTR-EGFP and tnw-MO2. (E) Percentage of embryos expressing EGFP following co-injection of CMV-tnwUTR-EGFP with tnw-5mmMO, tnw-MO1 or tnw-MO2. The error bars indicate the standard error. (TIF) [file pone.0069788.s009.tif]

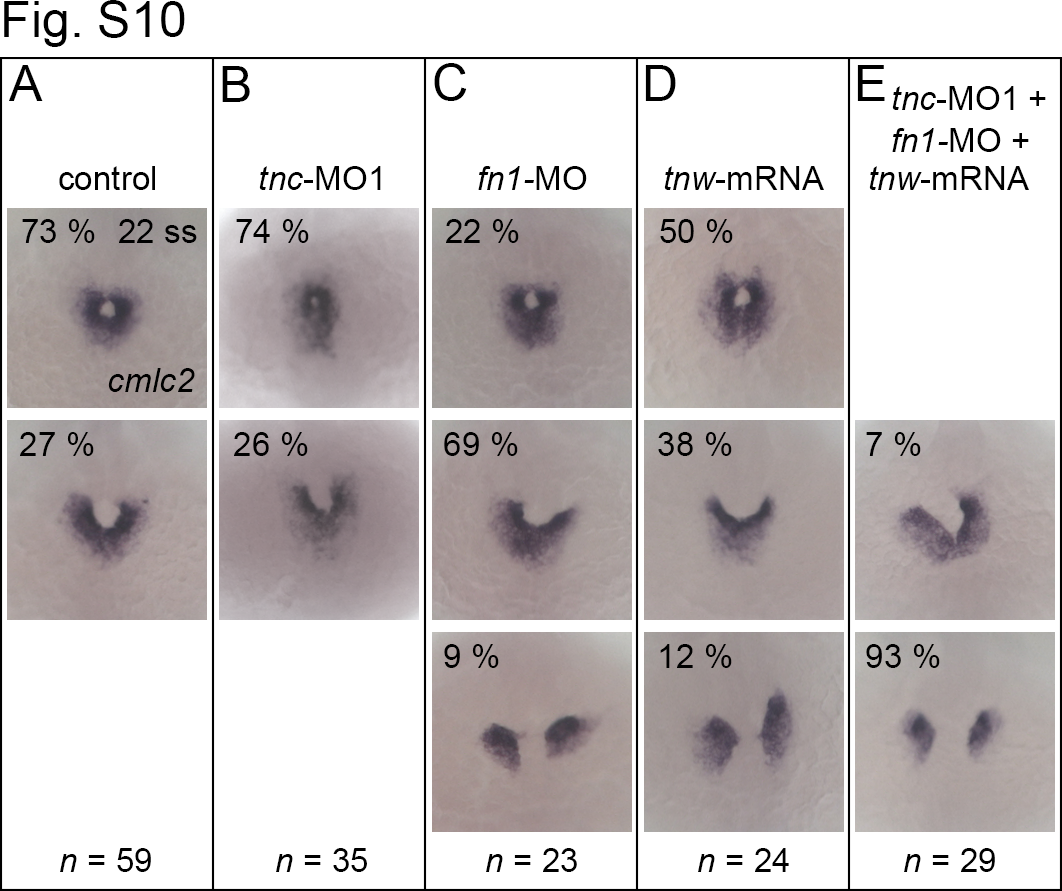

Supplement: Figure S10 — Knockdown of fn1 or tnc and overexpression of tnw results in cardia bifida in wild type embryos. Wild type embryos at the one-cell zygote stage were injected with 5 ng tnc-MO1 (B), 2.5 ng fn1-MO (C), 100 pg tnw mRNA (D), or a mixture of tnc-MO1, fn1-MO, and tnw mRNA (E), and incubated at 28.5°C. Un-injected embryos were used as a control (A). Embryos were harvested at the 22 ss and stained with cmlc2. (TIF) [file pone.0069788.s010.tif]
