# Supplementary material for: Low Temperature Mitigates Cardia Bifida in Zebrafish Embryos
Source: PLoS One. 2013 Jul 26;8(7):e69788. doi: 10.1371/journal.pone.0069788 (PMC3724881; doi:10.1371/journal.pone.0069788)
Supplement: Table S1 — Circulation defect of s1pr2as10 mutant embryos from different genotypes could be partially rescued when raised at 22.5°C. The statistics of the offspring derived from different genotypes of s1pr2as10 mutant raised at 28.5°C or 22.5°C. Genotype labeled with +/− or −/− indicated the heterozygous or homozygous s1pr2as10 mutants respectively. For example, among 213 embryos from heterozygous mutants intercross (+/− x +/−) raised at 28.5°C, 162 embryos showed normal wild-type phenotype (a), 46 embryos contained tail blisters phenotype and established no blood circulation (b), and 5 embryos contained tail blisters with normal circulation (c). Mendel ratio was calculated by number of embryos with tail blister phenotype divided by number of total embryos. Rescue of tail blister with circulation phenotype was observed in offspring derived from different genotypes of s1pr2as10 mutant raised at 22.5°C. Rescue percentage of tail blister with circulation phenotype was calculated by number of embryos showing tail blister with circulation phenotype divided by total number of embryos showing tail blister phenotype. (DOC) [file pone.0069788.s011.doc]

**Table S1. Circulation defect of *s1pr2^as10^*** mutant embryos from different genotypes could be partially rescued when raised at 22.5 ºC.

| genotype | temperature | total number of embryos:  a+b+c | wild-type like (a) | tail blisters without circulation (b) | tail blisters with  circulation (c) | ratio of tail blisters with circulation: c/(b+c) | Mendel ratio: (b+c)/(a+b+c) |
| --- | --- | --- | --- | --- | --- | --- | --- |
| +/- x +/- | 28.5 ºC | 213 | 162 | 46 | 5 | 10 % | 24 % |
|  | 22.5 ºC | 266 | 199 | 25 | 42 | 63 % | 25 % |
| +/- x -/- | 28.5 ºC | 112 | 59 | 50 | 3 | 6 % | 47 % |
|  | 22.5 ºC | 248 | 130 | 28 | 90 | 76 % | 48 % |
| -/- x -/- | 28.5 ºC | 52 | 0 | 47 | 5 | 10 % | 100 % |
|  | 22.5 ºC | 71 | 0 | 23 | 48 | 68 % | 100 % |

The statistics of the offspring derived from different genotypes of *s1pr2^as10^* mutant raised at 28.5 ºC or 22.5 ºC. Genotype labeled with +/- or -/- indicated the heterozygous or homozygous *s1pr2^as10^* mutants respectively. For example, among 213 embryos from heterozygous mutants intercross (+/- x +/-) raised at 28.5 ºC, 162 embryos showed normal wild-type phenotype (a), 46 embryos contained tail blisters phenotype and established no blood circulation (b), and 5 embryos contained tail blisters with normal circulation (c). Mendel ratio was calculated by number of embryos with tail blister phenotype divided by number of total embryos. Rescue of tail blister with circulation phenotype was observed in offspring derived from different genotypes of *s1pr2^as10^* mutant raised at 22.5 ºC. Rescue percentage of tail blister with circulation phenotype was calculated by number of embryos showing tail blister with circulation phenotype divided by total number of embryos showing tail blister phenotype.
