# Supplementary material for: Low Temperature Mitigates Cardia Bifida in Zebrafish Embryos
Source: PLoS One. 2013 Jul 26;8(7):e69788. doi: 10.1371/journal.pone.0069788 (PMC3724881; doi:10.1371/journal.pone.0069788)
Supplement: Table S2 — Up- and down-regulated genes in s1pr2as10 mutant at 22.5°C as determined by digital gene expression (DGE) analysis. Double-stranded cDNA from 22 ss-s1pr2as10 mutant embryos raised at 28.5°C and 22.5°C were synthesized for next generation sequencing. The Up- and down-regulated genes at 22.5°C were subsequently analyzed by pathway analysis according to Kyoto Encyclopedia of Genes and Genomes (KEGG) database. Several groups of genes were selected for further analysis. (DOC) [file pone.0069788.s012.doc]

**Table S2. Up- and down-regulated genes in *s1pr2^as10^* mutant at 22.5 ºC as determined by digital gene expression (DGE) analysis.**

| pathway | up-regulated | down-regulated |
| --- | --- | --- |
| Sphingolipid metabolism | sphingomyelin phosphodiesterase 4  (smpd4) | zgc:153896 |
| Leukocyte transendothelial migration |  | Dr.142307, Dr.118453, PDZ and LIM domain 7,  zgc:158463, Dr.79413, Dr.82248 |
| ECM-receptor interaction | collagen type XI alpha-2 (COL11A2),  fibronectin 1 (fn1) | secreted immunoglobulin domain 4 (sid4) |
| Oxidative phosphorylation | Dr.155447, Dr.78094 | Dr.76235 |
| Drug metabolism - cytochrome P450 |  | zgc:173961, zgc:101673 |

Double-stranded cDNA from 22 ss-*s1pr2^as10^* mutant embryos raised at 28.5 ºC and 22.5 ºC were synthesized for next generation sequencing. The Up- and down-regulated genes at 22.5 ºC were subsequently analyzed by pathway analysis according to Kyoto Encyclopedia of Genes and Genomes (KEGG) database. Several groups of genes were selected for further analysis.
